# UPR^mt^ scales mitochondrial network expansion with protein synthesis via mitochondrial import in *Caenorhabditis elegans*

**DOI:** 10.1038/s41467-020-20784-y

**Published:** 2021-01-20

**Authors:** Tomer Shpilka, YunGuang Du, Qiyuan Yang, Andrew Melber, Nandhitha Uma Naresh, Joshua Lavelle, Sookyung Kim, Pengpeng Liu, Hilla Weidberg, Rui Li, Jun Yu, Lihua Julie Zhu, Lara Strittmatter, Cole M. Haynes

**Affiliations:** 1 Department of Molecular, Cell and Cancer Biology, University of Massachusetts Medical School, Worcester, MA 01605 USA; 2 Department of Cellular and Physiological Sciences, Life Sciences Institute, University of British Columbia, Vancouver, BC Canada V6T 1Z3; 3 Electron Microscopy Core, University of Massachusetts Medical School, Worcester, MA 01605 USA

**Keywords:** Mitochondria, Energy metabolism, Caenorhabditis elegans, Chaperones

## Abstract

As organisms develop, individual cells generate mitochondria to fulfill physiological requirements. However, it remains unknown how mitochondrial network expansion is scaled to cell growth. The mitochondrial unfolded protein response (UPR^mt^) is a signaling pathway mediated by the transcription factor ATFS-1 which harbors a mitochondrial targeting sequence (MTS). Here, using the model organism *Caenorhabditis elegans* we demonstrate that ATFS-1 mediates an adaptable mitochondrial network expansion program that is active throughout normal development. Mitochondrial network expansion requires the relatively inefficient MTS in ATFS-1, which allows the transcription factor to be responsive to parameters that impact protein import capacity of the mitochondrial network. Increasing the strength of the ATFS-1 MTS impairs UPR^mt^ activity by increasing accumulation within mitochondria. Manipulations of TORC1 activity increase or decrease ATFS-1 activity in a manner that correlates with protein synthesis. Lastly, expression of mitochondrial-targeted GFP is sufficient to expand the muscle cell mitochondrial network in an ATFS-1-dependent manner. We propose that mitochondrial network expansion during development is an emergent property of the synthesis of highly expressed mitochondrial proteins that exclude ATFS-1 from mitochondrial import, causing UPR^mt^ activation.

## Abstract (web summary)

The mitochondrial network expands to accommodate cell growth, but how scaling occurs is unclear. Here, the authors show in *C. elegans* that ATFS-1 mitochondrial import is reduced when mitochondrial proteins are highly expressed, activating the unfolded protein response and causing expansion.

## Introduction

Mitochondria are double-membrane bound organelles localized throughout the cytosol of eukaryotic cells; mitochondria are required for diverse essential activities including ATP production, nucleotide and amino acid synthesis, as well as serving as hubs for programmed cell death and innate immune signaling^1^. Mitochondria comprised ~1100 proteins^2^ that are encoded by genes within the nuclear genome as well as within the mitochondrial genome (mtDNA) (12 in *Caenorhabditis elegans*; 13 in mammals). Nuclear-encoded mitochondrial proteins are synthesized on cytosolic ribosomes and subsequently imported into mitochondria via a series of import channels and machineries^3^. Although much is known about mitochondrial biogenesis, it remains unclear how expansion of the mitochondrial network scales with cell growth in order to meet the physiological needs of each cell type.

**Fig. 1:**
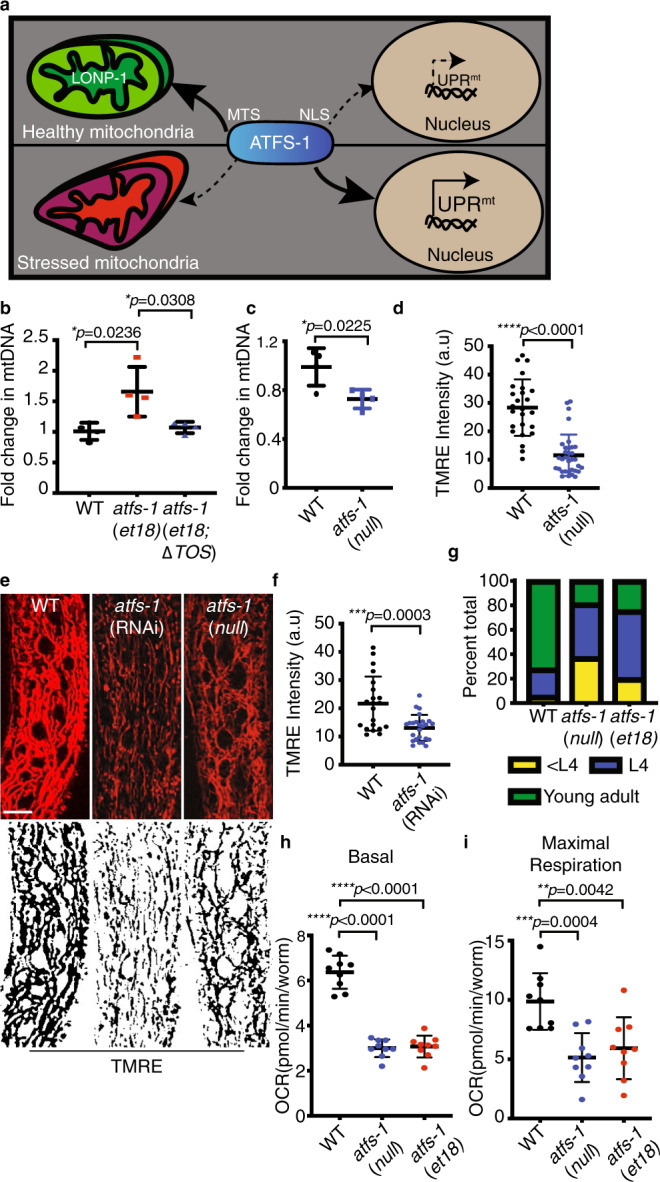
ATFS-1 regulates mitochondrial network expansion. **a** Schematic of ATFS-1 regulation. **b** Quantification of mtDNA in wild type, *atfs-1(et18)*, and *atfs-1(et18;ΔTOS)* as determined by qPCR. *N* = 4 biologically independent experiments. Error bars mean ± SD (two-tailed Student’s *t*-test). **c** Quantification of mtDNA in wild type and *atfs-1(null)*. *N* = 4 biologically independent experiments. Error bars mean ± SD (two-tailed Student’s *t*-test). **d** Quantification of TMRE intensity in the intestine of wild-type and *atfs-1(null)* worms. Experiments were repeated three biologically independent times with similar results. *N* = 21 worms (wild type), *N* = 33 worms *((atfs-1(null))*. Error bars mean ± SD (two-tailed Student’s *t*-test). a.u. arbitrary units. **e** TMRE staining of wild-type worms raised on control or *atfs-1*(RNAi) and *atfs-1(null)* worms. Skeleton-like binary backbone is presented (bottom). Experiments were repeated three biologically independent times with similar results. *N* = 21 worms. Scale bar 10 µm. **f** Quantification of TMRE intensity in the intestine of wild-type and *atfs-1*(RNAi) worms. Experiments were repeated three biologically independent times with similar results. *N* = 25 worms. Error bars mean ± SD (two-tailed Student’s *t*-test). a.u. Arbitrary units. **g** Developmental stages of 3-day-old wild-type, *atfs-1(et18)*, or *atfs-1(null)* worms. *N* = 546 worms(wild type), *N* = 597 worms (atfs-1(*et18*)) and 627 worms (*atfs-1(null)*). Experiments were repeated two biologically independent times with similar results. **h**, **i** Oxygen consumption rates (OCR) in wild type, *atfs-1(et18)*, and *atfs-1(null)*. Basal respiration (**h**), maximal respiration (**i**). *N* = 9 wells each containing ten worms. Error bars mean ± SD (two-tailed Student’s *t*-test). Experiment was performed three biologically independent times with similar results. Source data are provided as a Source Data file.

The UPR^mt^ is a mitochondrial-to-nuclear signal transduction pathway regulated by the transcription factor ATFS-1, which is required for development and longevity during mitochondrial dysfunction^4–6^. As ATFS-1 harbors a mitochondrial targeting sequence (MTS) and a nuclear localization sequence (NLS), its transcription activity is regulated by subcellular localization. If ATFS-1 is imported into mitochondria, it is degraded by the protease LONP-1^5^ (Fig. 1a). We previously showed that if a percentage of ATFS-1 fails to be imported into mitochondria, it traffics to the nucleus to activate a transcriptional response that includes mitochondrial chaperones^7,8^. Perturbations to oxidative phosphorylation (OXPHOS) or mitochondrial proteostasis activate the UPR^mt^ as both processes are required for mitochondrial protein import^9^. In turn, ATFS-1 induces transcription to promote survival, and recovery from mitochondrial stress^10^.

Here we show that ATFS-1 and the UPR^mt^ regulate an adaptable mitochondrial network expansion program that is active during *C. elegans* development. Worms lacking *atfs-*1 have reduced expression of numerous genes required for mitochondrial biogenesis, relatively small mitochondria and fewer mtDNAs. Conversely, constitutive UPR^mt^ activation results in increased mtDNA accumulation throughout development. We demonstrate that mitochondrial network expansion requires the weak MTS within ATFS-1, which allows the transcription factor to respond to reductions in mitochondrial protein import capacity. We find that impairment of S6 kinase results in impaired ATFS-1 activity due to mitochondrial accumulation of the transcription factor, while manipulations that increase TORC1-dependent protein synthesis activates the UPR^mt^, suggesting ATFS-1 responds to an increase in mitochondrial protein flux during development, to regulate mitochondrial network expansion. Consistent with this model, we demonstrate that over-expression of a single protein with a strong MTS is sufficient to expand the mitochondrial network in a manner dependent on ATFS-1.

## Results

### A role for ATFS-1 in mitochondrial network maintenance and expansion

We previously found that deleterious mtDNA heteroplasmy resulted in an *atfs-1*-dependent expansion of the mitochondrial network that was observed only when mitophagy was impaired^11^, suggesting a role for the UPR^mt^ in mediating mitochondrial biogenesis. To gain further insights into the relationship between ATFS-1 and mitochondrial biogenesis during mitochondrial stress, we examined mtDNA accumulation in *clk-1(qm30)* worms, which lack a component required for ubiquinone biogenesis and activates the UPR^mt^^12^. *clk-1(qm30)* worms harbored more mtDNAs than wild-type worms, suggesting UPR^mt^ activation leads to an increase in mtDNA quantity (Supplementary Fig. 1a).

*atfs-1(et18)* worms constitutively activate the UPR^mt^ due to an amino acid substitution in the MTS, which impairs import into mitochondria even in the absence of mitochondrial stress^13^. Impressively, *atfs-1(et18)* worms harbored more mtDNAs relative to wild-type worms in both the L3 (Supplementary Fig. 1b) and the L4 larval stages (Fig. 1b), suggesting that UPR^mt^ activation is sufficient to expand the mitochondrial network. Conversely, worms lacking the entire *atfs-1* open reading frame (*atfs-1(null)*)^14^ had reduced mtDNAs (Fig. 1c and Supplementary Fig. 1b). Moreover, tetramethylrhodamine, ethyl ester (TMRE) staining indicated that *atfs-1(null)* or *atfs-1* RNA interference (RNAi)-treated worms harbor fewer functional mitochondria relative to wild-type worms in intestinal cells (Fig. 1d–f). Combined these findings suggest the UPR^mt^ is actively involved in the maintenance and expansion of the mitochondrial network during development independent of exogenous mitochondrial stressors.

*atfs-1(null)* worms developed slowly and had reduced respiration relative to wild-type worms (Fig. 1g–i). *atfs-1(et18)* worms also developed slowly as previously documented^13^ (Fig. 1g). Surprisingly, despite the increased mtDNA quantity, *atfs-1(et18)* also respired less than wild-type worms (Fig. 1h, i). The reduction in respiratory capacity in the *atfs-1(et18)* strain was consistent with reduced TMRE staining in intestinal cells (Supplementary Fig. 1c, d). Thus, in the absence of the UPR^mt^, mtDNAs and functional mitochondrial are reduced, while continuous UPR^mt^ activation results in a partial expansion of the mitochondrial network that yields dysfunctional mitochondria.

### ATFS-1 mediates a mitochondrial expansion program during development

**Fig. 2:**
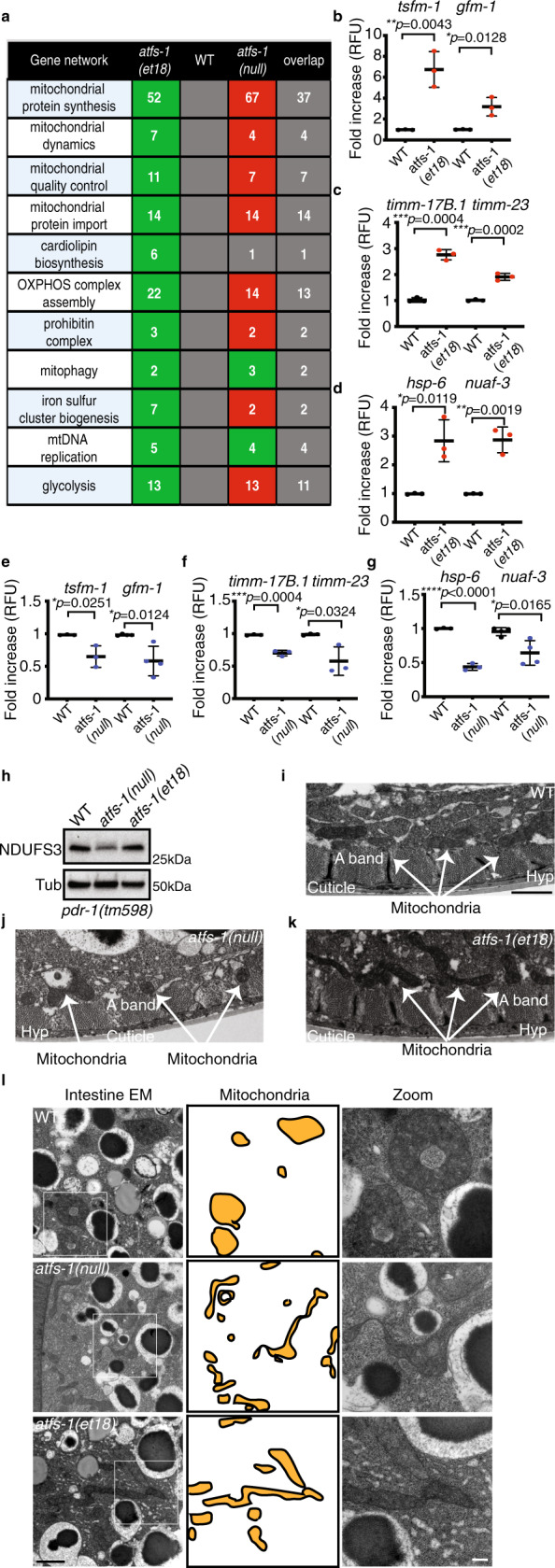
ATFS-1 mediates a mitochondrial network expansion program. **a** Number of differentially regulated genes induced in *atfs-1*(*et18*) worms relative to wild type (WT) or reduce in *atfs-1(null)* worms relative to wild type. Green, upregulated; red, downregulated; and the number of overlapping genes between *atfs-1(et18)* and *atfs-1(null)* worms are listed in the last column. **b**–**g** Transcript levels of translation elongation factor mitochondrial 1 (*tsfm-1*) and G elongation factor mitochondrial 1 (*gfm-1*) (**b**, **e**), translocase of inner mitochondrial membrane 17B.1 (*timm-17B.1*) and translocase of inner mitochondrial membrane-23 (*timm-23*) (**c**, **f**), heat shock protein-6 (*hsp-6*) and NADH:ubiquinone oxidoreductase complex assembly factor 3 (*nuaf-3*) (**d**, **g**) as determined by qRT-PCR in wild type and *atfs-1(et18)* (**b**–**d**) or in wild type and *atfs-1(null)* (**e**–**g**). *N* = 3 biologically independent experiments except for *gfm-1* (**e**) and *nuaf-3* (**g**) *N* = 4. Error bars mean ± SD (two-tailed Student’s *t*-test). RFU, relative fluorescence units. **h** SDS-PAGE immunoblots of lysates from *pdr-1(tm598*), *atfs-1(null);pdr-1(tm598)*, and *atfs-1(et18);pdr-1(tm598)* worms. NDUFS3 is a component of the NADH:Ubiquinone Oxidoreductase complex I and tubulin (Tub) was used as a loading control. *N* = 3 biologically independent experiments with similar results. **i**–**k**. Transmission electron microscopy of body wall muscle of wild type (**i**), *atfs-1(null)* (**j**), and *atfs-1(et18)* worms (**k**). Scale bar 1 µm. Representative images from five worms analyzed from two biologically independent experiments with similar results. **l** Transmission electron microscopy of intestinal cells from wild-type, *atfs-1(null)*, and *atfs-1(et18)* worms. Mitochondria are highlighted in the middle panel. Scale bar 1 µm (left) and 200 nm (right). Representative images from five worms analyzed from two biologically independent experiments with similar results. Source data are provided as a Source Data file.

To elucidate the role of ATFS-1 in mitochondrial expansion and homeostasis, we compared transcriptional profiles of wild-type, *atfs-1(null)*, and *atfs-1(et18*) worms during development in the absence of mitochondrial stress. As expected, *atfs-1(et18)* worms induced mitochondrial genes including the proteostasis components associated with the UPR^mt^ (Fig. 2a–d, Supplementary Fig. 2a–d, and Supplementary Data file 1). Furthermore, over 50 genes required for mitochondrial ribosome function were upregulated, as were genes required for mtDNA replication, and cardiolipin biosynthesis pathway genes that are essential for mitochondrial inner membrane synthesis. Lastly, genes required for both mitochondrial protein import and OXPHOS complex assembly were also upregulated.

Remarkably, most of the mitochondrial genes induced in the *atfs-1(et18)* strain were downregulated in the *atfs-1(null)* worms compared to wild-type worms (Figs.  2a, e–g, Supplementary Fig. 2b–d, and Supplementary Data files 2 and 3), consistent with less mtDNA and TMRE staining in intestinal cells (Fig. 1). Interestingly, the OXPHOS protein NDUFS3 was decreased in the *atfs-1(null)* worms, but unaffected in *atfs-1(et18)* worms (Fig. 2h and Supplementary Fig. 2e), both of which are consistent with the RNA-sequencing (RNA-seq) data in each strain where the mRNA encoding the NDUFS3 transcript was reduced in *atfs-1(null)* worms and unchanged in *atfs-1(et18)* worms (Supplementary Data files 4 and 5). To exclude potential effects of mitochondrial degradation via mitophagy, these studies were performed in a strain lacking *pdr-1* (Parkin)^15^. Interestingly, multiple mRNAs encoding tricarboxylic acid (TCA) cycle and OXPHOS components were repressed in *atfs-1(et18)* relative to wild-type worms (Supplementary Data file 5), consistent with our previous results where ATFS-1 limited transcription of highly expressed mitochondrial proteins during severe mitochondrial stress^8^. Lastly, although many mRNAs encoding mitochondrial proteins were expressed at lower levels in *atfs-1(null)* worms, mtDNA replication and mitophagy components were upregulated suggesting an alternative stress response(s) is induced in the absence of *atfs-1* (Fig. 2a).

Because of the alterations in mtDNA levels, TMRE, and transcription of mitochondrial components in *atfs-1* mutant worms, we visualized mitochondria via transmission electron microscopy. Impressively, mitochondria in *atfs-1(null)* worms were smaller and appeared defective in both intestine and muscle cells, along with pervasive muscle cell aberrations (Fig. 2i–l and Supplementary Fig. 2f). In contrast, mitochondria in *atfs-1(et18)* worms were elongated, which was especially apparent in the intestine (Fig. 2i–l and Supplementary Fig. 2f). As worms lacking *atfs-1* harbor small and dysfunctional mitochondria, and *atfs-1* is required for transcription of multiple genes required for mitochondrial biogenesis, our findings suggest that ATFS-1 regulates a mitochondrial expansion program. However, it should be considered that while ATFS-1 with an impaired MTS induces expression of mitochondrial biogenesis genes, it also impairs transcription of multiple TCA cycle and OXPHOS genes. These findings are similar to our previous results indicating that during severe mitochondrial dysfunction ATFS-1 bound the promoters of TCA cycle and OXPHOS genes and also limited their expression^8^.

### A weak MTS regulates ATFS-1, UPR^mt^, and mitochondrial network expansion

**Fig. 3:**
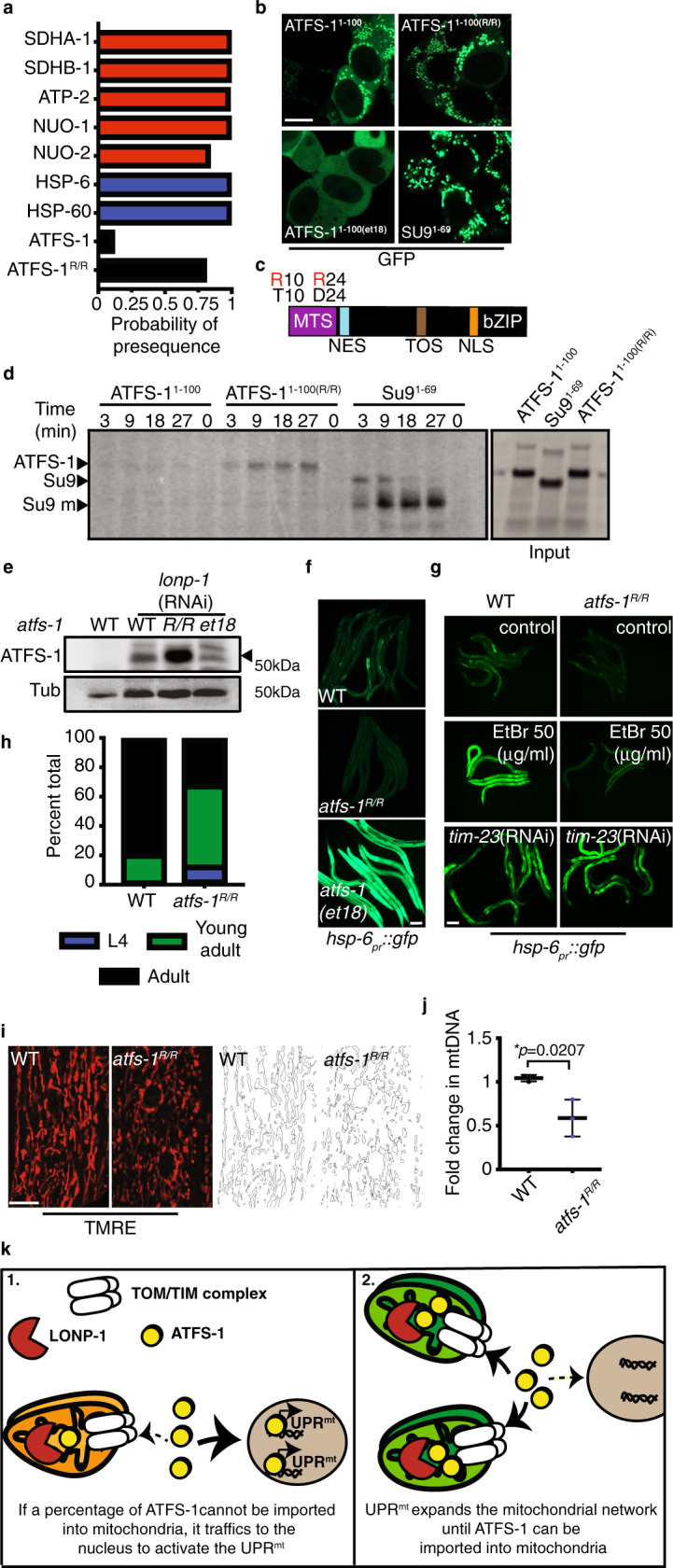
UPR^mt^ requires the weak MTS in ATFS-1. **a** Mitochondrial targeting sequence probability prediction using MitoFates. OXPHOS proteins (red), mitochondrial chaperones (blue), ATFS-1 (black). **b** HEK293T cells expressing ATFS-1^1–100^::GFP, ATFS-1^1-100(R/R)^::GFP, ATFS-1^1-100(et18)^::GFP or SU9^1–69^::GFP. Scale bar 10 µm. Experiment was repeated two biologically independent times with similar results. **c** ATFS-1 schematic highlighting ATFS-1^R/R^ amino acid substitutions. **d** In organelle import of radiolabeled ATFS-1^1–100^::GFP, ATFS-1^1-100(R/R)^::GFP and Su9^1–69^::GFP into isolated mitochondria. After the indicated time points, mitochondria were washed and analyzed by SDS-PAGE electrophoresis. ATFS-1, Su9, mature (m) are marked. *N* = 3 biologically independent experiments with similar results. **e** SDS-Page immunoblots of lysates from wild-type, *atfs-1*^*R/R*^, and *atfs-1(et18)* worms raised on control or *lonp-1*(RNAi). ATFS-1 is marked (‣) and tubulin (Tub) was used as a loading control. *N* = 3 biologically independent experiments with similar results. **f** *hsp-6*_*pr*_*::gfp* in wild-type, *atfs-1(et18)*, and *atfs-1*^*R/R*^ worms. Scale bar 0.1 mm. *N* = 3 biologically independent experiments with similar results. **g** *hsp-6*_*pr*_*::gfp* in wild-type and *atfs-1*^*R/R*^ worms raised on 50 µg/ml EtBr, control, or *timm-23*(RNAi). Scale bar 0.1 mm. *N* = 3 biologically independent experiments with similar results. **h** Developmental stages of 3-day-old wild-type or *atfs-1*^*R/R*^ worms. *N* = 158 worms (wild type) and *N* = 256 worms (*atfs-1*^*R/R*^). Experiments were repeated two biologically independent with similar results. **i** TMRE staining of wild-type and *atfs-1*^*R/R*^ worms. Skeleton-like binary backbone is presented (right). Scale bar 10 µm. Experiments were repeated three times with similar results. **j** Quantification of mtDNA in wild-type and *atfs-1*^*R/R*^ worms as determined by qPCR. *N* = 3 biologically independent experiments. Error bars mean ± SD (two-tailed Student’s *t*-test). **k** Proposed model for ATFS-1-mediated mitochondria expansion. Source data are provided as a Source Data file.

We next sought to determine how ATFS-1 is regulated, or excluded from mitochondria, during development. As ATFS-1 harbors an MTS along with a NLS, we have proposed that the UPR^mt^ is regulated by protein import capacity of the entire mitochondrial network^5^. ATFS-1 is predicted to have a relatively weak, or inefficient, MTS compared to other mitochondrial-targeted proteins such as mitochondrial chaperones and OXPHOS components^16–18^ (Fig. 3a). To compare the MTS strength of the OXPHOS protein ATP synthase subunit 9 (Su9) to ATFS-1, the N terminus of each was fused to green fluorescent protein (GFP) and expressed in HEK293T cells. As expected, both GFP-fusion proteins accumulated within mitochondria, but unlike Su9^(1-69)^::GFP, ATFS-1^(1-100)^::GFP fluorescence also accumulated within the cytosol, but to a lesser extent than that of ATFS-1^et18(1-100)^::GFP (Fig. 3b). In addition, import of ATFS-1^(1-100)^::GFP was limited compared to Su9^(1-69)^::GFP in an in vitro import assay (Fig. 3d), consistent with ATFS-1 harboring a weak MTS.

We hypothesized that the inefficient MTS allows ATFS-1 import and UPR^mt^ activation to be sensitized to conditions that impact mitochondrial import capacity including mitochondrial stress, total mitochondria, and potentially the flux of other proteins into mitochondria. Thus, we sought to generate a worm strain expressing ATFS-1 with a stronger, or more efficient, MTS. Amino acid substitutions of T10 and D24 to arginine are predicted to increase MTS strength^16^ (Fig.  3a, c). Similar to Su9^(1-69)^::GFP, ATFS-1^R/R(1-100)^::GFP only accumulated within mitochondria and not in the cytosol in HEK293T cells (Fig. 3b). Furthermore, more ATFS-1^R/R(1-100)^::GFP accumulated within mitochondria than ATFS-1^(1-100)^::GFP in an in vitro import assay, consistent with increased MTS strength (Fig. 3d).

Via CRISPR-Cas9, mutations were introduced at the endogenous *atfs-*1 locus to generate *atfs-1*^*R/R*^. We first examined accumulation of ATFS-1^R/R^ within mitochondria during normal development by raising worms on *lonp-1*(RNAi), which impairs ATFS-1 degradation within the matrix^5^. Strikingly, more ATFS-1^R/R^ accumulated within mitochondria compared to wild-type ATFS-1 or ATFS-1^et18^ during normal development (Fig. 3e). ATFS-1^R/R^ worms had reduced *hsp-6*_*pr*_*::gfp* fluorescence relative to wild-type or *atfs-1(et18)* worms during normal development (Fig. 3f and Supplementary Fig. 3a) and also had reduced expression of *hsp-6* and *timm-23* mRNAs (Supplementary Fig. 3b, c). ATFS-1^R/R^ also impaired UPR^mt^ activation caused by ethidium bromide (EtBr) exposure (Fig. 3g and Supplementary Fig. 3d). However, *timm-23*(RNAi), which impairs a component required for import of most proteins harboring N-terminal MTSs^9^, caused UPR^mt^ activation in both ATFS-1^R/R^ and wild-type worms (Fig. 3g and Supplementary Fig. 3d), indicating ATFS-1^R/R^ is a functional transcription factor likely impaired due to increased mitochondrial accumulation. Similar to worms lacking *atfs-1*, worms expressing ATFS-1^R/R^ developed slower (Fig. 3h) and exhibited a perturbed and fragmented mitochondrial network in both intestine and muscle cells along with a reduction in mtDNAs (Fig. 3i, j and Supplementary Fig. 3e–g).

Combined, these data suggest that ATFS-1 regulates a transcriptional program to expand mitochondrial biomass that is active throughout development and reliant on an inefficient MTS that confers sensitivity to conditions that impact mitochondrial import capacity. These findings suggest that during development a percentage of ATFS-1 cannot be imported into mitochondria of growing cells resulting in modest UPR^mt^ activation and mitochondrial network expansion (Fig. 3k).

### Interplay between components that regulate protein synthesis, mitochondrial import, and ATFS-1

Previous screens for components required for UPR^mt^ activation identified multiple regulators of growth-related protein synthesis including the insulin-like receptor *daf-2*, *rheb-1*, *mTOR* (*let-363)*, and *rsks-1* (S6 kinase)^12,19^. TORC1 regulates protein synthesis rates in response to diverse inputs including growth signals and cellular energetics^20^. Insulin-like signaling-TORC1 promotes protein synthesis by phosphorylating RSKS-1, which in turn, phosphorylates a ribosomal subunit^20^ (Supplementary Fig. 4a). Alternatively, the 5′-AMP-activated protein kinase (AMPK) limits TORC1 activity and protein synthesis when ATP levels are low^21,22^.

**Fig. 4:**
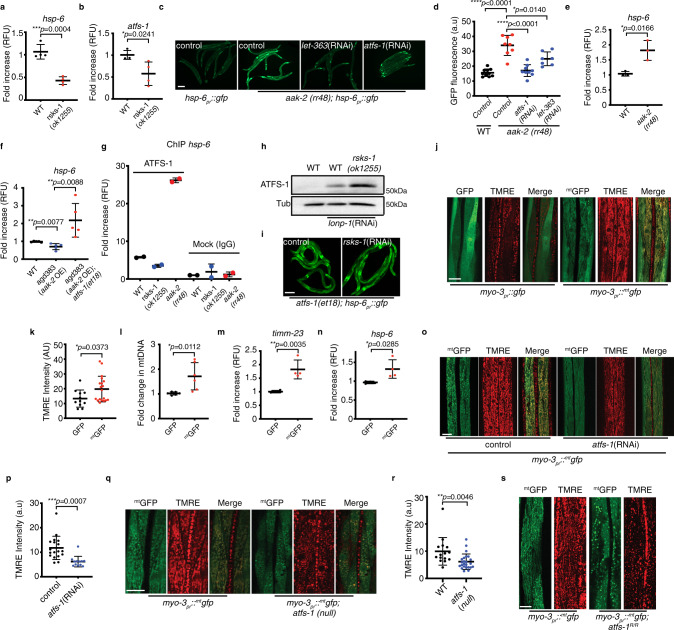
TORC1-mediated protein synthesis promotes ATFS-1 activation. **a**, **b** Transcript levels of heat shock protein-6 (*hsp-6*) (**a**) and of activated transcription factor stress-1 (*atfs-1*) (**b**) as determined by qRT-PCR in wild-type and *rsks-1(ok1255)* worms. *N* = 4 biologically independent experiments. Error bars mean ± SD (two-tailed Student’s *t*-test). RFU, relative fluorescence units. **c** *hsp-6*_*pr*_*::gfp* and *aak-2(rr48);hsp-6*_*pr*_*::gfp* worms raised on control, *let-363*, or *atfs-1* (RNAi). Scale bar 0.1 mm. Experiments were repeated three biologically independent times with similar results. **d** Quantification of GFP intensity in *hsp-6*_*pr*_*::gfp* (*N* = 10) and *aak-2(rr48);hsp-6*_*pr*_*::gfp* worms raised on control (*N* = 8 worms), *let-363* (*N* = 8 worms), or *atfs-1* (RNAi) (*N* = 7 worms). Error bars mean ± SD (two-tailed Student’s *t*-test). a.u., arbitrary units. **e**, **f** Transcript levels of heat shock protein-6 (*hsp-6*) as determined by qRT-PCR in wild-type and *aak-2(rr48)* worms *N* = 3 biologically independent experiments (**e**) and in wild type, *agd383*, and *agd383;atfs-1(et18)* strains *N* = 5 biologically independent experiments (**f**). Error bars mean ± SD (two-tailed Student’s *t*-test). RFU, relative fluorescence units. **g** ChIP of *hsp-6* promoter in wild-type, *rsks-1(ok1255)*, and *aak-2(rr48)* worms as measured by qRT-PCR. *N* = 2 biologically independent experiments. RFU, relative fluorescence units. **h** SDS-Page immunoblots of wild-type and *rsks-1(ok1255)* worms, raised on control or *lonp-1*(RNAi). Tubulin (Tub) was used as a loading control. *N* = 3 biologically independent experiments with similar results. **i** *atfs-1(et18);hsp-6*_*pr*_*::gfp* worms raised on control or *rsks-1*(RNAi). Scale bar 0.1 mm. Experiments were repeated three biologically independent times with similar results. **j** TMRE staining (red) of worms expressing *myo-3*_*pr*_*::gfp* or *myo-3*_*pr*_*::* ^*mt*^*gfp*. Scale bar 10 µm. Experiments were repeated three biologically independent times with similar results. **k** Quantification of TMRE intensity in muscle cells of worms expressing *myo-3*_*pr*_*::gfp* or *myo-3*_*pr*_*::* ^*mt*^*gfp*. *N* = 34 worms (*myo-3*_*pr*_*:: gfp*), *N* = 24 worms (*myo-3*_*pr*_*::* ^*mt*^*gfp)*. Error bars mean ± SD (two-tailed Student’s *t*-test). a.u., arbitrary units. **l** Quantification of mtDNA in *myo-3*_*pr*_*:: gfp* and *myo-3*_*pr*_*::* ^*mt*^*gfp* worms as determined by qPCR. *N* = 6 biologically independent experiments. Error bars mean ± SD (two-tailed Student’s *t*-test). **m**, **n** Transcript levels of translocase of inner mitochondrial membrane-23 (*timm-23*) (**m**) and of heat shock protein-6 (*hsp-6*) (**n**) as determined by qRT-PCR in *myo-3*_*pr*_*::gfp* and in *myo-3*_*pr*_*::* ^*mt*^*gfp* worms. *N* = 4 biologically independent experiments. Error bars mean ± SD (two-tailed Student’s *t*-test). RFU, relative fluorescence units. **o** TMRE staining of worms expressing *myo-3*_*pr*_*::* ^*mt*^*gfp* raised on control or *atfs-1*(RNAi). Scale bar 10 µm. Experiments were repeated three biologically independent times with similar results. **p** Quantification of TMRE intensity in muscle cells of worms expressing *myo-3*_*pr*_*::* ^*mt*^*gfp* raised on control or *atfs-1*(RNAi). *N* = 18 worms (control), *N* = 13 worms (*atfs-1*(RNAi)). Error bars mean ± SD (two-tailed Student’s *t*-test). a.u., arbitrary units. **q** TMRE staining of wild-type and *atfs-1*(*null*) worms expressing *myo-3*_*pr*_*::* ^*mt*^*gfp*. Scale bar 10 µm. Experiments were repeated three biologically independent times with similar results. **r** Quantification of TMRE intensity in muscle cells of wild-type, and *atfs-1(null)* worms. *N* = 15 worms (wild type), *N* = 24 worms (*atfs-1(null)*). Error bars mean ± SD (two-tailed Student’s *t*-test). a.u., arbitrary units. **s** TMRE staining of wild-type and *atfs-1*^*R/R*^ worms expressing *myo-3*_*pr*_*::* ^*mt*^*gfp*. Scale bar 10 µm. Experiments were repeated three biologically independent times with similar results. Source data are provided as a Source Data file.

As expected, DAF-2 inhibition impaired induction of *hsp-6*_*pr*_*::gfp* (Supplementary Fig. 4b)^23^. Furthermore, *hsp-6, atfs-1*, the mitochondrial elongation factors *gfm-1*, *tsfm-1*, and *timm-17* mRNAs were also reduced during normal development and during mitochondrial stress (Supplementary Fig. 4c–g) similar to what was observed in *atfs-1(null)* worms. Inhibition of TORC1 components *rheb-1*, *raga-1*, *mTOR*, and *rsks-1* also reduced *hsp-6*_*pr*_*::gfp* (Supplementary Fig. 4h–j), as well as *hsp-6* and *atfs-1*, *gfm-1*, *tsfm-1*, and *timm-17* mRNA levels, as did starvation (Fig. 4a, b and Supplementary Fig. 4k–p). Conversely, inhibition of AMPK, which increases TORC1 activity, resulted in increased *hsp-6* mRNA in an *atfs-1* and *mTOR*-dependent manner (Fig. 4c–e), whereas AMPK activation^24^ reduced *hsp-6* transcripts (Fig. 4f). Moreover, AMPK inhibition resulted in increased binding of ATFS-1 to the *hsp-6* promoter, suggesting increased nuclear localization of ATFS-1. Alternatively, the promoter occupancy of *hsp-6* by ATFS-1 was reduced in the *rsks-1(ok1255)* worms relative to wild-type worms (Fig. 4g). Combined, our results indicate that TORC1 and RSKS-1 are required for UPR^mt^ during development and suggest that the relatively high level of protein synthesis during development stimulates UPR^mt^ activity.

In mammals, TORC1 promotes protein synthesis by phosphorylating S6 kinase and 4EBP, which requires a TOR signaling (TOS) motif in each protein^25^. Interestingly, ATFS-1 also harbors a canonical TOS motif (-FEMDI-) (Fig. 3c), which we mutated at the endogenous locus to yield -AEMDI- (*atfs-1(∆TOS)*). UPR^mt^ activation was attenuated in *atfs-1(∆TOS)* worms relative to wild-type worms upon EtBr exposure (Supplementary Fig. 4q). Importantly, the TOS motif was also required for the increased *hsp-6*_*pr*_*::gfp* (Supplementary Fig. 4r) and mtDNA (Fig. 1b) in *atfs-1(et18)* worms suggesting the TOS motif promotes nuclear function of ATFS-1, similar to the TOS motif found in the transcription factor HIF-1α^26^. Interestingly, *hsp-6*_*pr*_*::gfp* induction caused by EtBr or complex III-deficiency (*isp-1(qm150)*^27^), was impaired further in *rsks-1(ok1255);atfs-1(∆TOS)* worms relative to either *atfs-1(∆TOS)* or *rsks-1(ok1255)* worms (Supplementary Fig. 4s–u), suggesting that RSKS-1 promotes UPR^mt^ activation independent of the TOS motif.

We examined the effect of *rsks-1* inhibition on expression and trafficking of ATFS-1. One possibility is that *rsks-1* inhibition simply reduces synthesis of ATFS-1 limiting its nuclear transcription activity. *rsks-1(ok1255)* worms had reduced *atfs-1* mRNA relative to wild-type worms (Fig. 4b). However, more ATFS-1 protein accumulated within mitochondria in *rsks-1(ok1255)* worms relative to wild-type worms (Fig. 4h) suggesting UPR^mt^ impairment in worms lacking RSKS-1 is due to mitochondrial accumulation of ATFS-1, similar to ATFS-1^R/R^ (Fig. 3e). Importantly, *rsks-1*(RNAi) did not reduce *hsp-6*_*pr*_*::gfp* in *atfs-1(et18)* worms with an impaired MTS (Fig. 4i) or when treated with *timm-23*(RNAi) (Supplementary Fig. 4v). Lastly, increased *hsp-6*_*pr*_*::gfp* persisted during starvation in both *atfs-1(et18)* worms and *timm-23*(RNAi) treated worms (Supplementary Fig. 4w, x). Combined, these findings suggest that the reduced *atfs-1* transcription in *rsks-*1-mutant worms is not the cause of impaired UPR^mt^ activation, rather the accumulation of ATFS-1 within mitochondria impairs *atfs-1-*dependent transcription.

As RSKS-1 is required for protein synthesis during cell growth, we hypothesized that the high rate of mitochondrial protein import during development may cause UPR^mt^ activation and mitochondrial network expansion. Interestingly, OXPHOS transcripts are among the most highly expressed mRNAs in worms (Supplementary Fig. 5a). In addition, *C. elegans* ribosome profiling data indicate that OXPHOS proteins are translated primarily during the early stages of worm development (L1,L2), and are reduced or absent by the L4 stage (Supplementary Fig. 5b). Interestingly, the ATFS-1 ribosome profile mirrors the OXPHOS profiles early in development and is also diminished at L4 (Supplementary Fig. 5c), consistent with the observation that the UPR^mt^ can only be activated by stress prior to the L4 stage^28^. Thus, we hypothesize that because of a weak MTS, a percentage of ATFS-1 is excluded from mitochondria during development by highly expressed mitochondrial proteins with stronger MTSs.

As a test of this model, we sought to determine the impact of overexpressing a single protein with a relatively strong MTS on the mitochondrial network. The mitochondrial network was examined in muscle cells of worms expressing GFP or ^mt^GFP via the strong *myo-3* promoter^29^. Importantly, ^mt^GFP harbors a relatively strong MTS (aa 1–24) from the enzyme aspartate aminotransferase (AST) (Supplementary Fig. 6a) and the *myo-3* promoter is expressed throughout development^30^ (Supplementary Fig. 6b). Strikingly, ^mt^GFP expression increased accumulation of functional mitochondria as determined by TMRE staining (Fig. 4j, k), mtDNA number (Fig. 4l), and *hsp-6*, *timm-17* and *timm-23* mRNAs (Fig. 4m–n and Supplementary Fig. 6c) despite ^mt^GFP being expressed at a lower level than GFP (Supplementary Fig. 6d, e). Expansion of TMRE staining by ^mt^GFP was impaired by *atfs-1*(RNAi) (Fig. 4o, p), *atfs-1(null)* (Fig. 4q, r), and in ATFS-1^R/R^ worms (Fig. 4s); however ^mt^GFP transcription was not affected by the *atfs-1(null)* allele (Supplementary Fig. 6f).

To determine whether the mitochondrial network expansion caused by expression of ^mt^GFP required nuclear trafficking of ATFS-1, we used CRISPR-Cas9 to introduce an amino acid substitution to perturb the NLS in ATFS-1 (R426A). *atfs-1*^*∆NLS*^ worms were unable to induce *hsp-6*_*pr*_*::gfp* or endogenous *hsp-6* transcripts during mitochondrial stress caused by *spg-7*(RNAi) consistent with impaired nuclear activity (Supplementary Fig. 6g, h). When raised on *lonp-*1(RNAi), ATFS-1^(∆NLS)^ accumulated within mitochondria like wild-type ATFS-1 (Supplementary Fig. 6i), indicating ATFS-1^∆NLS^ was expressed and processed similarly to wild-type ATFS-1. The *atfs-1*^*∆NLS*^ allele was crossed into *myo-3*_*pr*_*::*^*mt*^*GFP* worms to examine the effect on mitochondrial network expansion during development. Similar to *atfs-1*^R/R^ and *atfs-1(null)* worms, *atfs-1*^*∆NLS*^ worms had a severely perturbed mitochondrial network in muscle cells as determined by TMRE staining (Supplementary Fig. 6j). Thus, the NLS within ATFS-1 is essential for mitochondrial network expansion caused by expression of ^mt^GFP. Combined, these findings indicate that expression of a single protein with a strong MTS is sufficient to expand the mitochondrial network in an *atfs-1*-dependent manner.

## Discussion

The UPR^mt^ was discovered as a protective transcriptional response to unfolded protein accumulation within the mitochondria and OXPHOS perturbation^5,31–33^. Here, we demonstrate that ATFS-1 regulates a mitochondrial biogenesis or network expansion program that is basally active throughout normal development, which can be further activated by diverse mitochondrial perturbations. Developmental mitochondrial expansion required the inefficient MTS of ATFS-1, mTOR, and S6 kinase, suggesting an interplay between protein synthesis, mitochondrial protein import capacity, and nuclear activity of ATFS-1. We propose a model where the high levels of mitochondrial protein synthesis that occurs during development drives mitochondrial network expansion by excluding a percentage of ATFS-1 from mitochondrial import. In addition, network expansion continues until mitochondrial import capacity is sufficient to import ATFS-1 and terminate the UPR^mt^. These findings are conceptually similar to the endoplasmic reticulum expansion that occurs in response to increased protein flux via the UPR^ER^, which is regulated by IRE1 and XBP1^34^. We propose that as a function of mitochondrial import flux or mitochondrial protein processing, ATFS-1 scales mitochondrial network expansion with cell growth. Intriguingly, it was recently reported that mitochondrial metabolic proteins are prone to stalling within mitochondrial import channels under basal conditions in growing cells^35,36^, suggesting import or intra-mitochondrial protein processing can be overwhelmed during normal cell growth, which may result in ATFS-1 activation.

We previously found that during severe mitochondrial dysfunction ATFS-1 binds the promoters of many of the genes regulated by ATFS-1 during normal development including mitochondrial chaperones, mitochondrial protein import machinery, as well as mitochondrial ribosome component encoding genes. Intriguingly, the ATFS-1 chromatin immunoprecipitation (ChIP)-sequencing experiment also revealed that ATFS-1 bound the promoters many OXPHOS and TCA cycle genes. Surprisingly, ATFS-1 repressed or limited transcription of the OXPHOS and TCA cycle genes during mitochondrial stress. We suggested that repression of OXPHOS and TCA cycle transcription reduced the load of unfolded proteins in an already stressed mitochondria potentially allowing time for recovery^8^.

Ribosome profiling indicates that during normal development, the highly expressed OXPHOS proteins are translated during the L1–L2 developmental stages and mostly reduced by the L4 stage^30^. We suggest that it is the expression of these and other mitochondrial proteins that leads to developmental activation of ATFS-1 by limiting the mitochondrial import of ATFS-1, resulting in mitochondrial network expansion. Consistent with this model, expression of ^mt^GFP was sufficient to expand the muscle cell mitochondrial network in an *atfs-1*-dependent manner (Fig. 4j–s). If the mitochondrial network becomes perturbed during growth, reducing transcription of the most highly expressed substrates may prevent further damage and allow time for recovery.

These insights potentially inform on the discrepancies between *atfs-1(null)* worms and *atfs-1(et18)* worms where despite having increased mtDNAs and mitochondrial biogenesis machinery transcripts, the mitochondrial network in *atfs-1(et18)* worms is dysfunctional (Supplementary Fig. 1c, d). However, RNA-seq indicates that multiple TCA cycle and OXPHOS components are reduced in *atfs-1(et18)* worms relative to wild-type worms (Supplementary Data file 5). It is important to note that the *atfs-1(et18)* allele was isolated in a screen to identify mutations that confer resistance to high levels of statins^13^. Although the processes affected in *atfs-1(et18)* worms that confer statin resistance are unknown, the strain develops slowly and has a shortened lifespan relative to wild-type worms^13^. One potential reason is that in the *atfs-1(et18)* strain, ATFS-1 is not regulated by mitochondrial import capacity and remains constitutively active where in addition to increasing numerous genes that promote mitochondrial biogenesis also represses OXPHOS and TCA cycle component transcription. This uncoupling, or deregulation, may result in the generation of defective mitochondria.

In conclusion, we propose that ATFS-1 responds to the high levels of mitochondrial protein import that occur during development. The relatively inefficient MTS of ATFS-1 matches insulin receptor (DAF-2) and S6 kinase-dependent synthesis of mitochondrial proteins to a transcriptional program that expands the mitochondrial network effectively scaling mitochondrial biogenesis with cell growth. However, if mitochondrial function is perturbed, ATFS-1 mitochondrial import is further perturbed causing additional nuclear accumulation where it reduces transcription of OXPHOS and TCA cycle components^8^ reducing the burden on the mitochondrial protein folding and complex assembly machinery. Upon network recovery, developmental mitochondrial network expansion continues until mitochondrial protein import capacity is sufficient to import ATFS-1.

## Methods

### Worms, plasmids, and staining

The reporter strain *hsp-6*_*pr*_*::gfp* for visualizing UPR^mt^, and the *myo-3*_*pr*_*::gfp* and the *myo-3*_*pr*_*::*^*mt*^*gfp* for visualization of mitochondrial mass and *atfs-1(null)* worms have been previously described^14,32,33^. The MTS in the *myo-3*_*pr*_*::*^*mt*^*gfp* is the first 24 amino acids from the enzyme AST from *Coturnix japonica* (1-MALLQSRLLLSAPRRAAATARASS-24) fused to GFP. The *atfs-1(et18)* strain was a gift from Marc Pilon. N2(wild type), *isp-1(qm150)*, *rsks-1(ok1255)*, and *daf-2(e1370)* were obtained from the Caenorhabditis Genetics Center (Minneapolis, MN).

The *atfs-1*^*R/R*^(*cmh16*), the *atfs-1(∆TOS)*(*cmh17*), and the *atfs-1(∆NLS)(cmh18)* strains were generated via CRISPR-Cas9 in *hsp-6*_*pr*_*::gfp* worms as described^14^. The *atfs-1(∆TOS)* was generated in both the wild-type worms as well as in the *atfs-1(et18)* strain. The crRNAs (IDT) were co-injected with purified Cas9 protein, tracrRNA (Dharmacon), repair templates (IDT), and the pRF4::rol-6(su1006) plasmid as described^37,38^. The crRNAs and repair templates used in this study are listed in Supplementary Data file 6. The pRF4::rol-6 (su1006) plasmid was a gift from Craig Mello^39^. The ATFS-1^1–100^::GFP expressing plasmid was previously described^5^. The ATFS-1^1-100(R4C)^::GFP and the ATFS-1^1-100(T10R, D24R)^::GFP were generated by introducing mutations to yield the described amino acid substitutions in the ATFS-1^1–100^::GFP expressing plasmid. The Su9 of the F0-ATPase (SU9)^1–69^::GFP PQCXIP expression plasmid was a gift from Xuejun Jiang.

Worms were raised HT115 strain of *Escherichia coli* and RNAi performed as described^40^. EtBr and TMRE experiments were performed by synchronizing and raising worms on plates previously soaked with M9 buffer containing EtBr or 2 µM TMRE.

Worms were analyzed at the L4 larvae stage except for EtBr-treated worms that led to developmental arrest. EtBr-treated worms were analyzed at the same time as the control.

### Protein analysis and antibodies

Synchronized worms were raised on plates with control(RNAi) or *lonp-1*(RNAi) to the L4 stage prior to harvesting. Whole worm lysate preparation was previously described^33^. Antibodies against α-tubulin were purchased from Calbiochem (CP06), GFP and for NDUFS3 from Abcam (ab6556 and ab14711, respectively). Antibodies for ATFS-1 were previously described^5^. All antibodies were diluted 1 : 2000, except for ATFS-1, which was diluted 1 : 1000. Immunoblots were visualized using ChemiDoc XRS + system (Bio-Rad). All western blot experiments were performed at least three times.

### mtDNA quantification

mtDNA quantification was performed using a quantitative PCR (qPCR)-based method similar to previously described assays^41^. Twenty to 30 worms were collected in 30 µl of lysis buffer (50 mM KCl, 10 mM Tris-HCl pH 8.3, 2.5 mM MgCl_2_, 0.45% NP-40, 0.45% Tween 20, 0.01% gelatin, with freshly added 200 µg/ml proteinase K) and frozen at −80 °C for 20 min prior to lysis at 65 °C for 80 min. Relative quantification was used for determining the fold changes in mtDNA between samples. 1 µl of lysate was used in each triplicate qPCR reaction. qPCR was performed using the Thermo Scientific SyBr Green Maxima Mix and the MyiQ2 Two-Color Real-Time PCR Detection System (Bio-Rad Laboratories). Primers that specifically amplify mtDNA are listed in Supplementary Data file 6. Primers that amplify a non-coding region near the nuclear-encoded *ges-1* gene were used as a control. mtDNA was harvested from synchronized worms at the L4 stage. All qPCR results have been repeated at least three times and performed in triplicates. A Student’s *t*-test was employed to determine the level of statistical significance.

### RNA isolation and qRT-PCR

RNA isolation and quantitative reverse-transcriptase PCR (qRT-PCR) analysis were previously described^11^. Worms were synchronized by bleaching, raised on HT115 *E. coli* and harvested at the L4 stage. Total RNA was extracted from frozen worm pellets using RNA STAT (Tel-Test) and 500 ng RNA was used for cDNA synthesis with qScript™ cDNA SuperMix (QuantaBio). qPCR was performed using iQ™ SYBR® Green Supermix (Bio-Rad Laboratories). qPCR primers are listed in Supplementary Data file 6. All qPCR results were repeated at least 3 times and performed in triplicates. A two-tailed Student’s *t*-test was employed to determine the level of statistical significance.

### Oxygen consumption

Oxygen consumption was measured using a Seahorse XFe96 Analyzer at 25 °C similar to that described previously^42^. In brief, L4 worms were transferred onto empty plates and allowed to completely digest the remaining bacteria for 1 h, after which ten worms were transferred into each well of a 96-well microplate containing 180 µl M9 buffer. Basal respiration was measured for a total of 30 min, in 6 min intervals that included a 2 min mix, a 2 min time delay, and a 2 min measurement. To measure respiratory capacity, 15 µM carbonyl cyanide-4-(trifluoromethoxy)phenylhydrazone was injected, the oxygen consumption rate reading was allowed to stabilize for 6 min, then measured for five consecutive intervals. Mitochondrial respiration was blocked by adding 40 mM Sodium azide. Each measurement was considered one technical replicate.

### Cultured cells and imaging

HEK293T cells were transfected with 0.5 µg of the expression plasmids: SU9^1–69^::GFP with ATFS-1^1-100^::GFP, ATFS-1^1-100(R/R)^::GFP, and ATFS-1^1-100(et18)^::GFP via Lipofectamine. The cells were imaged 16 h post transfection.

### RNA-seq and differential expression analysis

cDNA libraries were constructed with standard Illumina P5 and P7 adapter sequences. The cDNA libraries were run on an Illumina HiSEq2000 instrument with single-read 50 bp. RNA reads were then aligned to WBcel235/ce11 reference genome and differential gene expression analysis was performed with edgeR^43^. Differences in gene expression between *atfs-1(et18)* and *atfs-1(null)* compared to wild type are listed in Supplementary Data files 7 and 8, respectively. Data were deposited in GEO accession no. GSE114951

### Analysis of worm development

Worms were synchronized via bleaching and allowed to develop on HT115 bacteria plates for 3 days at 20 °C. Developmental stage was quantified as a percentage of the total number of animals. Each experiment was preformed three times. For the comparison of wild-type and *atfs-1(null)* worms; *N* = 162 (wild type), and 282 (*atfs-1(null)*). For the comparison of wild type to *atfs-1*^*R/R*^ worms; *N* = 158 (wild type) and *N* = 256 (*atfs-1*^*R/R*^).

### Statistics

All experiments were performed at least three times yielding similar results and comprised of biological replicates. The sample size and statistical tests were chosen based on previous studies with similar methodologies and the data met the assumptions for each statistical test performed. No statistical method was used in deciding sample sizes. No blinded experiments were performed, and randomization was not used. For all figures, the mean ± SD is represented unless otherwise noted. Prism 8 (GraphPad) is used for statistical analysis and graph creation.

### Microscopy

*C. elegans* were imaged using either a Zeiss AxioCam 506 mono camera mounted on a Zeiss Axio Imager Z2 microscope or a Zeiss AxioCam MRc camera mounted on a Zeiss SteREO Discovery.V12 stereoscope. Images with high magnification (×63) were obtained using the Zeiss ApoTome.2. Exposure times were the same in each experiment. Cell cultures were imaged with the Zeiss LSM800 microscope. With the Zen 2.3 Blue software. All images are representatives of more than three images. Quantification of fluorescent intensity as well as creating binary skeleton-like structures were done with the Fiji software (Version 2.0.0-rc-69/1.52p)^44^.

### Gene set enrichment analysis

The OXPHOS gene set was downloaded from WormBase Ontology Browser^45^. mRNA abundance was measured and ranked by reads per kilobase per million reads from RNA-seq data. Pre-ranked gene set enrichment analysis was performed with GSEA3.0 software with ‘classical’ scoring^46^.

### Transmission electron microscopy

L4 larvae were transferred to 2.5% glutaraldehyde in 0.1 M Sodium Cacodylate buffer pH 7.2 for 10 min. The tail and head of each worm were dissected out and the main body was transferred to fresh 2.5% glutaraldehyde in 0.1 M Sodium Cacodylate buffer and kept at 4 °C overnight. Samples were processed and analyzed at the University of Massachusetts Medical School Electron Microscopy core facility according to standard procedures. Briefly, the samples were rinsed three times in the same fixation buffer and post-fixed with 1% osmium tetroxide for 1 h at room temperature. Samples were then washed three times with ddH_2_O for 10 min and then dehydrated through a graded ethanol series of 20% increments, before two changes in 100% ethanol. Samples were then infiltrated first with two changes of 100% Propylene Oxide and then with a 50%/50% propylene oxide/SPI-Pon 812 resin mixture. The following day, five changes of fresh 100% SPI-Pon 812 resin were performed before the samples were polymerized at 68 °C in flat pre-filled embedding molds. The samples were then reoriented, and thin sections (~70 nm) were placed on copper support grids and contrasted with Lead citrate and Uranyl acetate. Sections were examined using a CM10 TEM with 100 Kv accelerating voltage and images were captured using a Gatan TEM CCD camera.

### Ribosome profiling data analysis

Ribosome profiling sequencing data were downloaded from the NCBI Sequence Read Archive (SRA) (http://www.ncbi.nlm.nih.gov/sra/) under accession number SRA055804. Data were analyzed as previously described^30^. Data analysis was done with the help of Unix-based software tools. First, the quality of raw sequencing reads was determined by FastQC (version 0.11.9)^47^. Reads were then filtered according to quality via FASTQ for a mean Phred base calling quality score above 30^48^. Filtered reads were mapped to the *C. elegans* reference genome (Wormbase WS275) using BWA (version 0.7.5) and SAM files were converted into BAM files by SAMtools (version 0.1.19). Coverage data for specific genes (including 5′-untranslated region (UTR), exons and 3′-UTR) were calculated by SAMtools and coverage data for each gene was plotted using R (version 3.5.2)^49^.

### Mitochondria isolation and in vitro protein import

Cells (budding yeast W303) were grown to logarithmic phase in YPD (1% yeast extract, 2% peptone, 2% glucose), collected by centrifugation, and washed once with water. Cells were then resuspended in 0.1 M Tris pH 9.4, 10 mM dithiothreitol, and incubated for 20 min at 30 °C. Cell walls were disturbed by incubation in 1.2 M sorbitol, 20 mM K_2_HPO_4_ pH 7.4, 1% zymolyase for 1 h at 30 °C. Dounce homogenization was used to lyse the cells in 0.6 M sorbitol, 10 mM Tris pH 7.4, 1 mM EDTA, fatty acid free 0.2% bovine serum albumin (BSA), and 1 mM phenylmethylsulfonyl fluoride (PMSF). Mitochondria were then isolated by differential centrifugation as described previously^50^ and resuspended in SEM buffer (0.25 M sucrose, 10 mM MOPS-KOH pH 7.2, and 1 mM EDTA).

The coupled Transcription/Translation system (T7 Quick for PCR DNA, Promega) was used to express ATFS-1 from a PCR template. Precursor proteins (ATFS-1^1–100^::GFP, ATFS-1^1-100(R/R)^::GFP, and Su9^1–69^::GFP) were synthesized in reticulocyte lysate in the presence of [35 S]methionine (T7 Quick for PCR DNA, Promega). Import into isolated mitochondria was performed in import buffer (3% (w/v) BSA, 250 mM sucrose, 80 mM KCl, 5 mM methionine, 5 mM MgCl_2_, 2 mM KH_2_PO_4_, 10 mM MOPS-KOH, pH 7.2, 4 mM NADH, 2 mM ATP, 5 mM creatine phosphate, 0.1 mg/ml creatine kinase) at 25 °C. The import reaction was stopped on ice or by addition of AVO (8 μM antimycin A, 20 μM oligomycin, 1 μM valinomycin). To dissipate Δψ, AVO was added before the import experiment. Samples were treated with 25 μg/ml proteinase K for 15 min on ice, following by treatment with 2 mM PMSF for 5 min on ice. Mitochondrial were washed twice with SEM buffer and analyzed by electrophoresis on SDS-polyacrylamide gel electrophoresis. The dried gel was exposed to a phospho screener, which was then scanned using a Typhoon Trio scanner (Amersham).

### Chromatin immunoprecipitation

ChIP assays for ATFS-1 were performed as previously described^8^. Synchronized worms were cultured in liquid and harvested at early L4 stage by sucrose flotation. The worms were lysed via Teflon homogenizer in cold phosphate-buffered saline (PBS) with protease inhibitors (Roche). Cross-linking of DNA and protein was performed by treating the worms with 1.85% formaldehyde with protease inhibitors for 15 min. Glycine was added to a final concentration of 125 mM and incubated for 5 min at room temperature to quench the formaldehyde. The pellets were resuspended twice in cold PBS with protease inhibitors. Samples were sonicated in a Bioruptor (Diagenode) for 15 min at 4 °C on high intensity (30 s on and 30 s off). Samples were transferred to microfuge tubes and spun at 15,000 × *g* for 15 min at 4 °C. The supernatant was precleaned with pre-blocked ChIP-grade Pierce™ magnetic protein A/G beads (Thermo Scientific) and then incubated with ATFS-1 antibody or Mouse mAb IgG1 Isotype Control (Cell Signaling Technology, G3A1) rotating overnight at 4 °C. The antibody-DNA complex was precipitated with protein A/G magnetic beads or protein A sepharose beads (Invitrogen). After washing, the crosslinks were reversed by incubation at 65 °C overnight. The samples were then treated with RNaseA at 37 °C for 1.5 h followed by proteinase K at 55 °C for 2 h. Lastly, the immunoprecipitated and input DNA were purified with ChIP DNA Clean & Concentrator (Zymo Research, D5205) and used as templates for qPCR.

### Reporting summary

Further information on research design is available in the Nature Research Reporting Summary linked to this article.

## Supplementary information

Supplementary Information

Supplementary Data file 1

Supplementary Data file 2

Supplementary Data file 3

Supplementary Data file 4

Supplementary Data file 5

Supplementary Data file 6

Supplementary Data file 7

Supplementary Data file 8

Description of additional supplementary files

Reporting Summary

## Data Availability

The data reported in this paper have been deposited in the Gene Expression Omnibus (GEO) database (accession number GSE114951). Data also available from the corresponding author upon reasonable request. Source data are provided with this paper.

## Source data

Source Data

## References

[1] Tan, J. X. & Finkel, T. Mitochondria as intracellular signaling platforms in health and disease. *J. Cell Biol*. **219**, 10.1083/jcb.202002179 (2020). 10.1083/jcb.202002179 PMC7199861 32320464

[2] Rath, S. et al. MitoCarta3.0: an updated mitochondrial proteome now with sub-organelle localization and pathway annotations. *Nucleic Acids Res*. 10.1093/nar/gkaa1011 (2020). 10.1093/nar/gkaa1011 PMC7778944 33174596

[3] Pfanner N, Warscheid B, Wiedemann N (2019). Mitochondrial proteins: from biogenesis to functional networks. Nat. Rev. Mol. Cell Biol..

[4] Berendzen KM (2016). Neuroendocrine coordination of mitochondrial stress signaling and proteostasis. Cell.

[5] Nargund AM, Pellegrino MW, Fiorese CJ, Baker BM, Haynes CM (2012). Mitochondrial import efficiency of ATFS-1 regulates mitochondrial UPR activation. Science.

[6] Sorrentino V (2017). Enhancing mitochondrial proteostasis reduces amyloid-beta proteotoxicity. Nature.

[7] Haynes CM, Yang Y, Blais SP, Neubert TA, Ron D (2010). The matrix peptide exporter HAF-1 signals a mitochondrial UPR by activating the transcription factor ZC376.7 in *C. elegans*. Mol. Cell.

[8] Nargund AM, Fiorese CJ, Pellegrino MW, Deng P, Haynes CM (2015). Mitochondrial and nuclear accumulation of the transcription factor ATFS-1 promotes OXPHOS recovery during the UPR(mt). Mol. Cell.

[9] Pfanner, N., Warscheid, B. & Wiedemann, N. Mitochondrial proteins: from biogenesis to functional networks. *Nat. Rev. Mol. Cell Biol*. 10.1038/s41580-018-0092-0 (2019). 10.1038/s41580-018-0092-0 PMC6684368 30626975

[10] Shpilka T, Haynes CM (2018). The mitochondrial UPR: mechanisms, physiological functions and implications in ageing. Nat. Rev. Mol. Cell Biol..

[11] Lin YF (2016). Maintenance and propagation of a deleterious mitochondrial genome by the mitochondrial unfolded protein response. Nature.

[12] Baker BM, Nargund AM, Sun T, Haynes CM (2012). Protective coupling of mitochondrial function and protein synthesis via the eIF2alpha kinase GCN-2. PLoS Genet..

[13] Rauthan M, Ranji P, Aguilera Pradenas N, Pitot C, Pilon M (2013). The mitochondrial unfolded protein response activator ATFS-1 protects cells from inhibition of the mevalonate pathway. Proc. Natl Acad. Sci. USA.

[14] Deng, P. et al. Mitochondrial UPR repression during *Pseudomonas aeruginosa* infection requires the bZIP protein ZIP-3. *Proc. Natl Acad. Sci. USA*, 10.1073/pnas.1817259116 (2019). 10.1073/pnas.1817259116 PMC6442607 30850535

[15] Narendra D, Tanaka A, Suen DF, Youle RJ (2008). Parkin is recruited selectively to impaired mitochondria and promotes their autophagy. J. Cell Biol..

[16] Fukasawa Y (2015). MitoFates: improved prediction of mitochondrial targeting sequences and their cleavage sites. Mol. Cell Proteomics.

[17] Melber A, Haynes CM (2018). UPR(mt) regulation and output: a stress response mediated by mitochondrial-nuclear communication. Cell Res..

[18] Rolland SG (2019). Compromised mitochondrial protein import acts as a signal for UPR(mt). Cell Rep..

[19] Haynes CM, Petrova K, Benedetti C, Yang Y, Ron D (2007). ClpP mediates activation of a mitochondrial unfolded protein response in *C. elegans*. Dev. Cell.

[20] Saxton RA, Sabatini DM (2017). mTOR signaling in growth, metabolism, and disease. Cell.

[21] Dillin A, Crawford DK, Kenyon C (2002). Timing requirements for insulin/IGF-1 signaling in C. elegans. Science.

[22] Zhang, Y. et al. Neuronal TORC1 modulates longevity via AMPK and cell nonautonomous regulation of mitochondrial dynamics in *C. elegans*. *Elife* **8**, 10.7554/eLife.49158 (2019). 10.7554/eLife.49158 PMC6713509 31411562

[23] Gatsi R (2014). Prohibitin-mediated lifespan and mitochondrial stress implicate SGK-1, insulin/IGF and mTORC2 in *C. elegans*. PLoS ONE.

[24] Mair W (2011). Lifespan extension induced by AMPK and calcineurin is mediated by CRTC-1 and CREB. Nature.

[25] Schalm SS, Blenis J (2002). Identification of a conserved motif required for mTOR signaling. Curr. Biol..

[26] Land SC, Tee AR (2007). Hypoxia-inducible factor 1alpha is regulated by the mammalian target of rapamycin (mTOR) via an mTOR signaling motif. J. Biol. Chem..

[27] Feng J, Bussiere F, Hekimi S (2001). Mitochondrial electron transport is a key determinant of life span in *Caenorhabditis elegans*. Dev. Cell.

[28] Durieux J, Wolff S, Dillin A (2011). The cell-non-autonomous nature of electron transport chain-mediated longevity. Cell.

[29] Labrousse AM, Zappaterra MD, Rube DA, van der Bliek AM (1999). C. elegans dynamin-related protein DRP-1 controls severing of the mitochondrial outer membrane. Mol. Cell.

[30] Stadler M, Artiles K, Pak J, Fire A (2012). Contributions of mRNA abundance, ribosome loading, and post- or peri-translational effects to temporal repression of *C. elegans* heterochronic miRNA targets. Genome Res..

[31] Zhao Q (2002). A mitochondrial specific stress response in mammalian cells. EMBO J..

[32] Benedetti C, Haynes CM, Yang Y, Harding HP, Ron D (2006). Ubiquitin-like protein 5 positively regulates chaperone gene expression in the mitochondrial unfolded protein response. Genetics.

[33] Yoneda T (2004). Compartment-specific perturbation of protein handling activates genes encoding mitochondrial chaperones. J. Cell Sci..

[34] Gass JN, Gunn KE, Sriburi R, Brewer JW (2004). Stressed-out B cells? Plasma-cell differentiation and the unfolded protein response. Trends Immunol..

[35] Ordureau A (2020). Global landscape and dynamics of Parkin and USP30-dependent ubiquitylomes in iNeurons during mitophagic dignaling. Mol. Cell.

[36] Phu L (2020). Dynamic regulation of mitochondrial import by the ubiquitin system. Mol. Cell.

[37] Paix A, Folkmann A, Rasoloson D, Seydoux G (2015). High efficiency, homology-directed genome editing in *Caenorhabditis elegans* using CRISPR-Cas9 ribonucleoprotein complexes. Genetics.

[38] Dokshin GA, Ghanta KS, Piscopo KM, Mello CC (2018). Robust genome editing with short single-stranded and long, partially single-stranded DNA donors in *Caenorhabditis elegans*. Genetics.

[39] Mello CC, Kramer JM, Stinchcomb D, Ambros V (1991). Efficient gene transfer in *C. elegans*: extrachromosomal maintenance and integration of transforming sequences. EMBO J..

[40] Rual JF (2004). Toward improving *Caenorhabditis elegans* phenome mapping with an ORFeome-based RNAi library. Genome Res..

[41] Valenci I, Yonai L, Bar-Yaacov D, Mishmar D, Ben-Zvi A (2015). Parkin modulates heteroplasmy of truncated mtDNA in *Caenorhabditis elegans*. Mitochondrion.

[42] Koopman M (2016). A screening-based platform for the assessment of cellular respiration in *Caenorhabditis elegans*. Nat. Protoc..

[43] Robinson MD, McCarthy DJ, Smyth GK (2010). edgeR: a Bioconductor package for differential expression analysis of digital gene expression data. Bioinformatics.

[44] Schindelin J (2012). Fiji: an open-source platform for biological-image analysis. Nat. Methods.

[45] Grove C (2018). Using WormBase: a genome biology resource for *Caenorhabditis elegans* and related nematodes. Methods Mol. Biol..

[46] Subramanian A (2005). Gene set enrichment analysis: a knowledge-based approach for interpreting genome-wide expression profiles. Proc. Natl Acad. Sci. USA.

[47] Wingett SW, Andrews S (2018). FastQ Screen: a tool for multi-genome mapping and quality control. F1000Res.

[48] Blankenberg D (2010). Manipulation of FASTQ data with Galaxy. Bioinformatics.

[49] Ihaka R, Gentleman R (1996). R: A language for data analysis and graphics. J. Comput. Graph. Stat..

[50] Weidberg, H. & Amon, A. MitoCPR-A surveillance pathway that protects mitochondria in response to protein import stress. *Science* **360**, 10.1126/science.aan4146 (2018). 10.1126/science.aan4146 PMC6528467 29650645

