# Supplementary material for: UPRmt scales mitochondrial network expansion with protein synthesis via mitochondrial import in Caenorhabditis elegans
Source: Nat Commun. 2021 Jan 20;12:479. doi: 10.1038/s41467-020-20784-y (PMC7817664; doi:10.1038/s41467-020-20784-y)
Supplement: Supplementary file 1 — Supplementary Information [file 41467_2020_20784_MOESM1_ESM.pdf]

SUPPLEMENTARY INFORMATION

UPR^{mt} scales mitochondrial network expansion with protein synthesis via mitochondrial import in *Caenorhabditis elegans*

Tomer Shpilka[1], YunGuang Du[1], Qiyuan Yang[1], Andrew Melber[1], Nandhitha U. Naresh[1], Joshua Lavelle[1], Sookyung Kim[1], Pengpeng Liu[1], Hilla Weidberg[2], Rui Li[1], Jun Yu[1], Lihua Julie Zhu[1], Lara Strittmatter[3] and Cole M. Haynes[1*]

[1]Department of Molecular, Cell and Cancer Biology, University of Massachusetts Medical School, Worcester, MA 01605, USA.
[2]Department of Cellular and Physiological Sciences, Life Sciences Institute, University of British Columbia, Vancouver, BC V6T 1Z3, Canada.
[3]Electron Microscopy Core, University of Massachusetts Medical School, Worcester, MA 01605, USA.

[*]Correspondence: cole.haynes@umassmed.edu

Supplementary Fig. 1

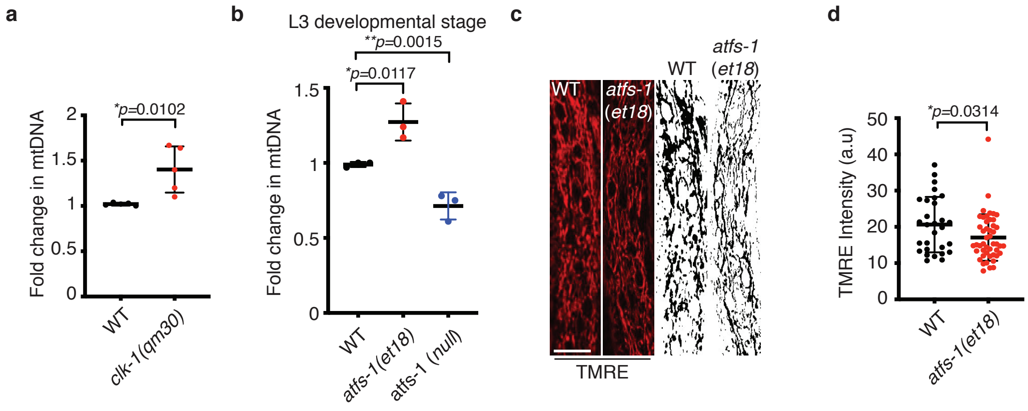

Supplementary Fig. 1. *clk-1(qm30)* harbor more mtDNAs than wildtype worms.

a. Quantification of mtDNA in wildtype, and *clk-1(qm30)* worms as determined by qPCR. N=4 biologically independent experiments. Error bars mean +/- s.d, (Two tailed Student's *t*-test).

b. Quantification of mtDNA in wildtype, *atfs-1(et18)* and *atfs-1(null)* worms at the L3 larvae stage, as determined by qPCR. N=3 biologically independent experiments. Error bars mean +/- s.d, (Two tailed Student's *t*-test).

c. TMRE staining of wildtype and *atfs-1(et18)* worms. Skeleton-like binary backbone is presented (right). Scale bar 10 μm. Experiments were repeated 3 biologically independent times with similar results.

d. Quantification of TMRE intensity of panel 1b. n=29 worms (wildtype) n=47 worms (*atfs-1(et18)*), Error bars mean +/- s.d (Two tailed Student's *t*-test). a.u- arbitrary units.

Source data are provided as a Source Data file.

Supplementary Fig. 2

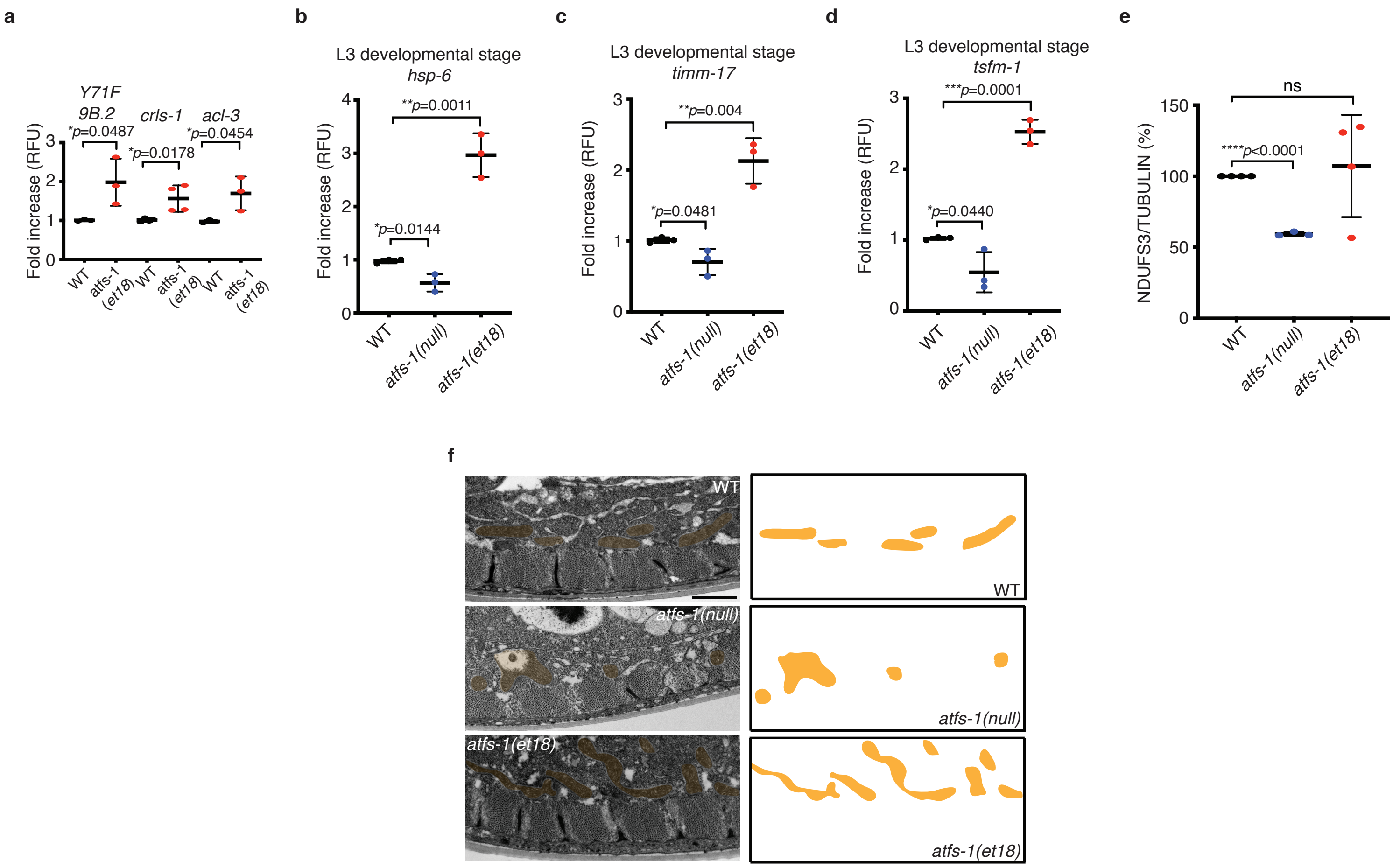

Supplementary Fig. 2. mRNAs encoding for mitochondrial proteins are differentially expressed in *atfs-1(et18)* and *atfs-1(null)* worms relative to wildtype worms.

a. Transcript levels of the TAM41 mitochondrial translocator assembly and maintenance homolog (*Y71F9B.2*), acyltransferase-like 3 (*acl-3*) and cardiolipin synthase homolog (*crls-1*) as determined by qRT-PCR in wildtype and *atfs-1(et18)* worms. N=3 or N=4 for *crls-1* biologically independent experiments. Error bars mean +/- s.d, (Two tailed Student's *t*-test). RFU – relative fluorescence units.

b-d. Transcript levels of heat shock protein 6 (*hsp-6*) (**b**), translocase of inner mitochondrial membrane 17B.1 (*timm-17B.1*) (**c**) and translation elongation factor mitochondrial 1 (*tsfm-1*) (**d**) as determined by qRT-PCR in wildtype, *atfs-1(null)* and *atfs-1(et18)* L3 worms. N=3 biologically independent experiments. Error bars mean +/- s.d, (Two tailed Student's *t*-test). RFU – relative fluorescence units.

e. Quantification of NDUFS3 levels relative to TUBULIN in *pdr-1(tm598)*, atfs-*1(null);pdr-1(tm598)* and *atfs-1(et18);pdr-1(tm598)* strains. N=3 *(atfs-1(null))*, N=4 *(wildtype and atfs-1(et18))* biologically independent experiments. Error bars mean +/- s.d, (Two tailed Student's *t*-test). %-percentage.

f. Transmission electron microscopy of body wall muscles of wildtype, *atfs-1(null)* and *atfs-1(et18)*. Mitochondria are highlighted in yellow. Scale bar 1 μm.

Source data are provided as a Source Data file.

Supplementary Fig. 3

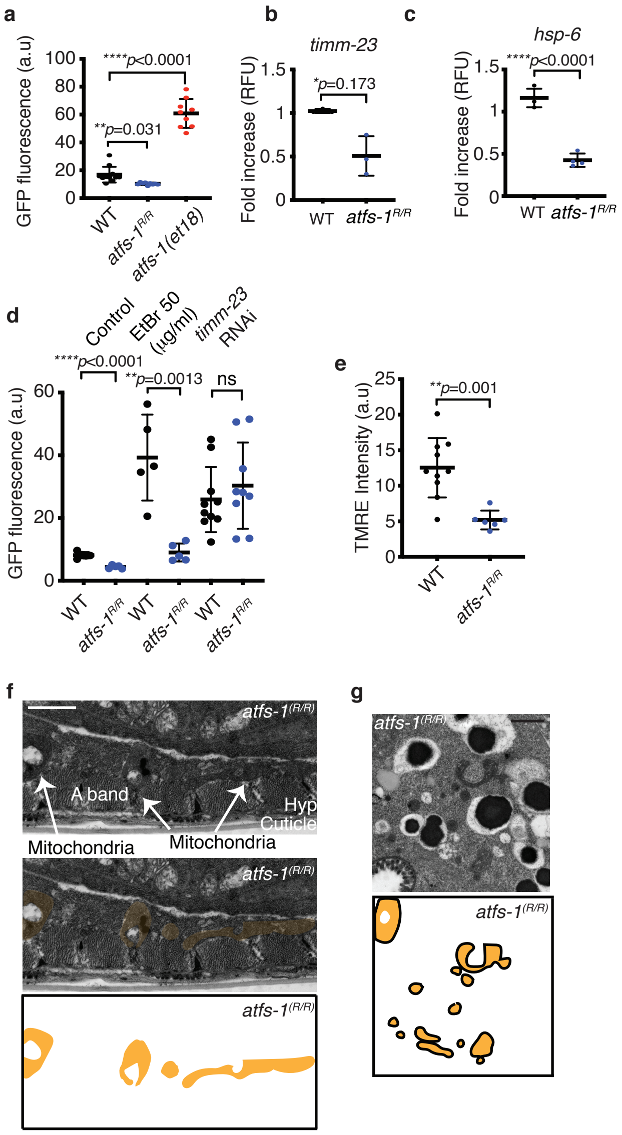

Supplementary Fig. 3. Increasing the strength of the ATFS-1 MTS inhibits the UPR^{mt} and perturbs the mitochondrial network.

a. Quantification of GFP intensity in wildtype, *atfs-1*$^{R/R}$ and *atfs-1(et18)* worms expressing *hsp-6*$_{pr}$*::gfp*. N=11 worms(wildtype), N=9 worms(*atfs-1*$^{R/R}$ and *atfs-1(et18)*). Error bars mean +/- s.d, (Two tailed Student's *t*-test). a.u- arbitrary units.

b-c. Transcript levels of translocase of inner mitochondrial membrane-23 (*timm-23*) (**b**) and of heat shock protein-6 (*hsp-6*) (**c**) as determined by qRT-PCR in wildtype and *atfs-1*$^{R/R}$ worms. N=4 (*hsp-6*), N=3 (*timm-23*) biologically independent experiments. Error bars mean +/- s.d, (Two tailed Student's *t*-test). RFU – relative fluorescence units.

d. Quantification of GFP intensity in wildtype and *atfs-1*$^{R/R}$ worms expressing *hsp-6*$_{pr}$*::gfp* and raised on control(RNAi), *timm-23*(RNAi) or 50 µg/ml EtBr. N=5 worms for control and EtBr. For *timm-23*(RNAi) N=10,9 worms (wildtype and *atfs-1*$^{R/R}$, respectively). Error bars mean +/- s.d, (Two tailed Student's *t*-test). a.u- arbitrary units.

e. Quantification of TMRE intensity in wildtype and *atfs-1*$^{R/R}$ worms. N=10,6 worms (wildtype and *atfs-1*$^{R/R}$, respectively). Error bars mean +/- s.d, (Two tailed Student's *t*-test). a.u- arbitrary units.

f. Transmission electron microscopy of body wall muscle cells of *atfs-1*$^{R/R}$ worms (wildtype control in Figure 2i). Mitochondria are highlighted in yellow. Scale bar 1 µm. Representative images from 5 worms analyzed by EM. N=2 biologically independent experiments with similar results.

g. Transmission electron microscopy of intestinal cells of *atfs-1*$^{R/R}$ worms. Mitochondria are highlighted in yellow. (wildtype control in Figure 2l). Scale bar 1 µm. Representative images from 5 worms analyzed by EM.

Source data are provided as a Source Data file.

Supplementary Fig. 4

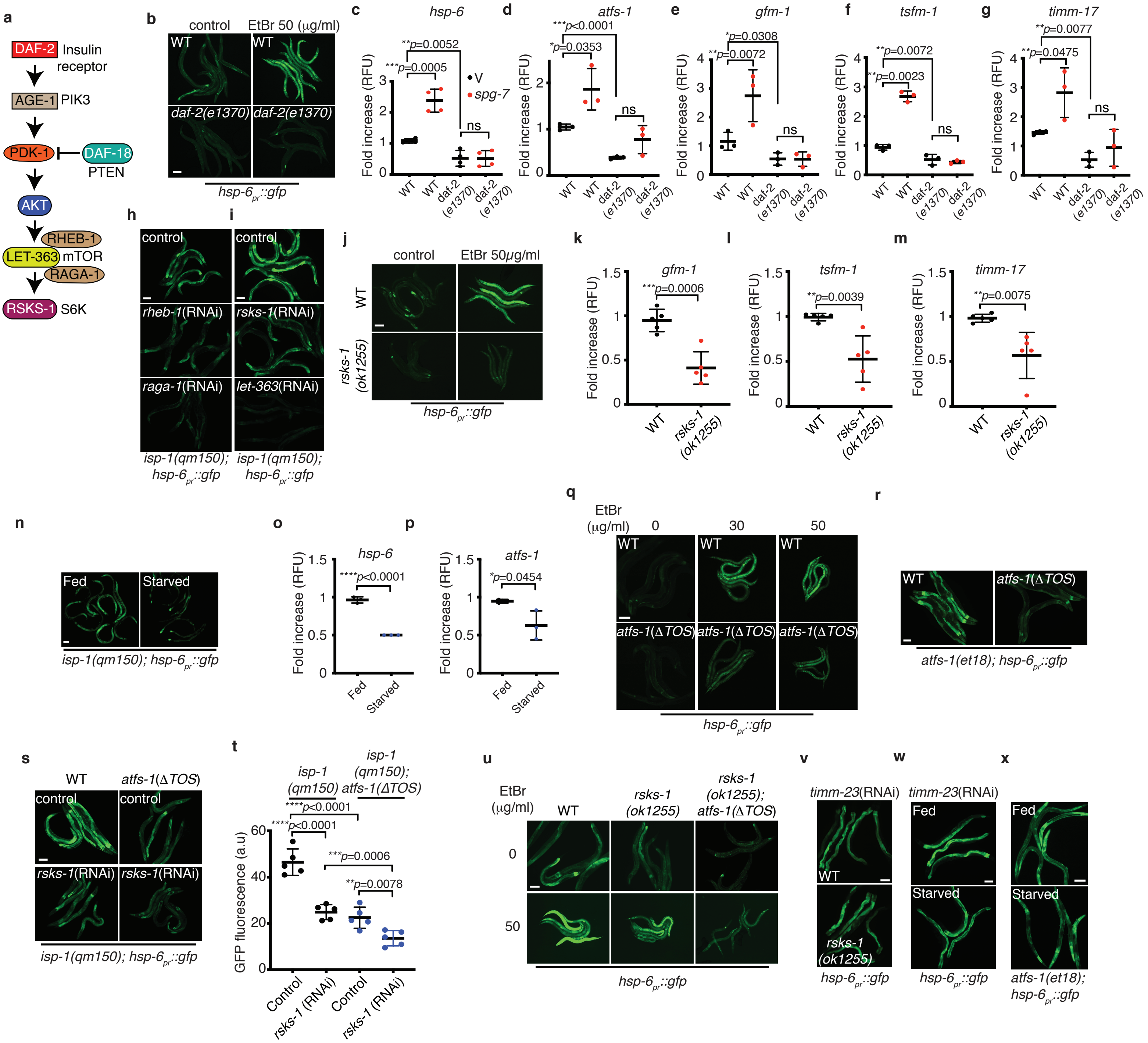

Supplementary Fig. 4. TORC1 regulates the UPR^{mt}

a. Schematic of the *C. elegans* insulin-like signaling and TORC1 pathway. Mammalian homologs included. The small GTPase Rheb is required for TORC1 activity, while the GTPase RAGA-1 regulates TORC1 activity in response to amino acid availability.

b. *hsp-6*$_{pr}$*::gfp* and *daf-2(e1370);hsp-6*$_{pr}$*::gfp* worms raised on control or 50 µg/ml EtBr at 20°C. Scale bar 0.1 mm. Experiments were repeated 3 biologically independent times with similar results.

c-g. Transcript levels of heat shock protein-6 (*hsp-6*) (**c**), activated transcription factor stress-1 (*atfs-1*) (**d**), G elongation factor mitochondrial 1 (*gfm-1*) (**e**), translation elongation factor mitochondrial 1 (*tsfm-1*) (**f**) and translocase of inner mitochondrial membrane 17B.1 (*timm-17B.1*) (**g**) as determined by qRT-PCR in wildtype and *daf-2(e1370)* strains raised on control(RNAi) or *spg-7*(RNAi) at 20°C. N=4(*hsp-6*), N=3(*atfs-1*, *gfm-1*, *tsfm-1*, *timm-17B.1*) biologically independent experiments. Error bars mean +/- s.d. (Two tailed Student's *t*-test). RFU – relative fluorescence units.

h. Photomicrographs of *isp-1(qm150)*;*hsp-6*$_{pr}$*::gfp* worms raised on control, *rheb-1* or *raga-1*(RNAi). Scale bar 0.1 mm. Experiments were repeated 3 biologically independent times with similar results.

i. Photomicrographs of *isp-1(qm150)*;*hsp-6*$_{pr}$*::gfp* worms raised on control, *rsks-1* or *let-363*(RNAi). Scale bar 0.1 mm. Experiments were repeated 3 biologically independent times with similar results.

j. Photomicrographs of *hsp-6*$_{pr}$*::gfp* and *rsks-1(ok1255);hsp-6*$_{pr}$*::gfp* worms raised on 0 or 50 µg/ml EtBr. Scale bar 0.1 mm. Experiments were repeated 3 biologically independent times with similar results.

k-m. Transcript levels of G elongation factor mitochondrial 1 (*gfm-1*) (**k**), translation elongation factor mitochondrial 1 (*tsfm-1*) (**l**) and translocase of inner mitochondrial membrane 17B.1 (*timm-17B.1*) (**m**) as determined by qRT-PCR in wildtype and *rsks-1(ok1255)* strains. N=4(*gfm-1*), N=5(*tsfm-1*, *timm-17B.1*) biologically independent experiments. Error bars mean +/- s.d. (Two tailed Student's *t*-test). RFU – relative fluorescence units.

n. Photomicrographs of *isp-1(qm150)*;*hsp-6$_{pr}$::gfp* worms raised to the L4 stage and starved for 1h. Scale bar 0.1 mm. Experiments were repeated 3 biologically independent times with similar results.

o-p. Transcript levels of heat shock protein-6 (*hsp-6*) (**o**) and activated transcription factor stress-1 (*atfs-1*) (**p**) as determined by qRT-PCR in worms raised to L4 and starved for 1h. N=3 biologically independent experiments. Error bars mean +/- s.d. (Two tailed Student's t-test). RFU – relative fluorescence units.

q. *hsp-6$_{pr}$::gfp* and *atfs-1(ΔTOS);hsp-6$_{pr}$::gfp* worms raised on 0,30 or 50 µg/ml EtBr. Scale bar 0.1 mm. Experiments were repeated 3 biologically independent times with similar results.

r. *atfs-1(et18)*;*hsp-6$_{pr}$::gfp* and *atfs-1(et18,ΔTOS)*;*hsp-6$_{pr}$::gfp* worms. Scale bar 0.1mm. Experiments were repeated 3 biologically independent times with similar results.

s. *isp-1(qm150)*;*hsp-6$_{pr}$::gfp* and *isp-1(qm150)*;*atfs-1(ΔTOS)*;*hsp-6$_{pr}$::gfp* worms raised on control or *rsks-1*(RNAi). Scale bar 0.1 mm. Experiments were repeated 3 biologically independent times with similar results.

t. Quantification of the experiment in panel s. N=5 worms. Error bars mean +/- s.d, (Two tailed Student's *t*-test). a.u- arbitrary units.

u. *hsp-6*$_{pr}$*::gfp, rsks-1(ok1255);hsp-6*$_{pr}$*::gfp* and *rsks-1(ok1255);atfs-1(∆TOS);hsp-6*$_{pr}$*::gfp* worms raised on 0 or 50 µg/ml EtBr. Scale bar 0.1 mm. Experiments were repeated 3 biologically independent times with similar results.

v. Photomicrographs of wildtype and *rsks-1(ok1255);hsp-6*$_{pr}$*::gfp* worms raised on *timm-23*(RNAi). Scale bar 0.1 mm. Experiments were repeated 3 biologically independent times with similar results.

w. Photomicrographs of *hsp-6*$_{pr}$*::gfp* worms raised on *timm-23*(RNAi) to the L4 stage and starved for 24 h. Scale bar 0.1 mm. Experiments were repeated 3 biologically independent times with similar results.

x. Photomicrographs of *atfs-1(et18)*;*hsp-6*$_{pr}$*::gfp* worms raised to the L4 stage and starved for 24 h. Scale bar 0.1 mm. Experiments were repeated 3 biologically independent times with similar results.

Source data are provided as a Source Data file.

Supplementary Fig. 5

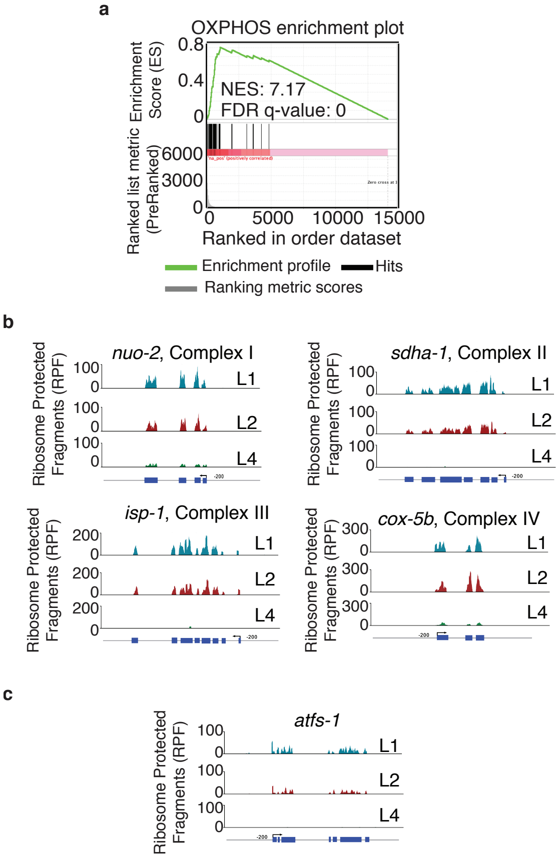

Supplementary Fig. 5. Translation of OXPHOS complex components primarily occurs during the early stages of worm development, similar to ATFS-1.

a. Gene Set Enrichment Analysis (GSEA) comparing the expression of OXPHOS genes to all other genes in *C. elegans*. Gene expression abundances were measured and ranked by reads per kilobase per million reads (RPKM) from the RNA-seq data of WT L4 worms. Each black line represents an OXPHOS gene and each white line represents genes that are not OXPHOS genes.

b. Ribosome protected fragments (RPF) profile of four representative OXPHOS complex subunits (*nuo-2* – complex I, *sdha-1* – complex 2, *isp-1* – complex 3 and *cox-5b* -complex 4) at different larval developmental stages (L1, L2 and L4).

c. Ribosome protected fragments (RPF) profile of *atfs-1* at the L1, L2, and L4 larval developmental stages.

Supplementary Fig. 6

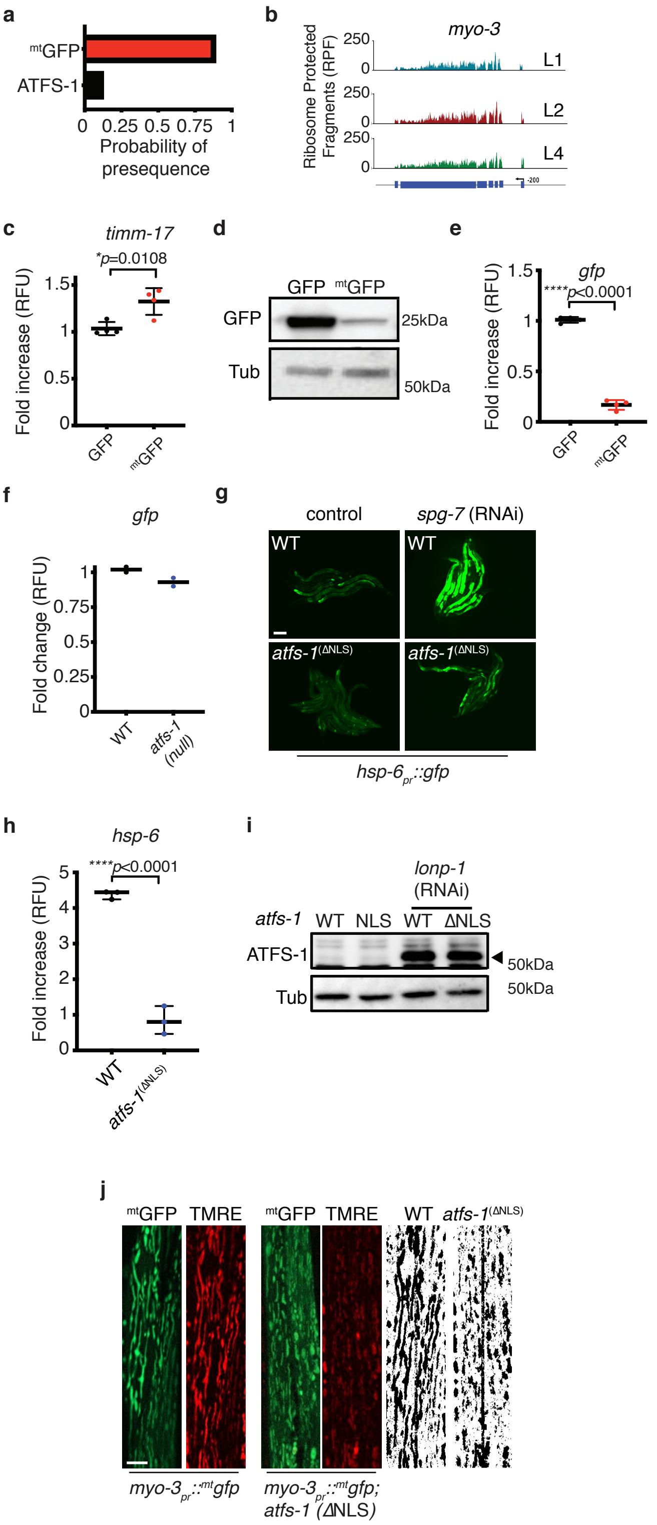

Supplementary Fig. 6. Mitochondrial expansion is regulated by mitochondrial protein import

a. Mitochondrial targeting sequence probability prediction using MitoFates. The MTS of aspartate aminotransferase (amino acids 1-24) (AST) fused to GFP (red) and ATFS-1 (black) are presented.

b. Ribosome protected fragments (RPF) profile of *myo-3* at the L1, L2, and L4 larval developmental stages.

c. Transcript levels of translocase of inner mitochondrial membrane-17 (*timm-17*) as determined by qRT-PCR in *myo-3*$_{pr}$*::gfp* and in *myo-3*$_{pr}$*::* ^{mt}*gfp* worms. N=4 biologically independent experiments. Error bars mean +/- s.d, (Two tailed Student's *t*-test). RFU – relative fluorescence units.

d. Immunoblots of GFP and Tubulin in *myo-3*$_{pr}$*::gfp* and *myo-3*$_{pr}$*::* ^{mt}*gfp* expressing worms.

e. Transcript levels of green fluorescent protein (*gfp*) as determined by qRT-PCR in *myo-3*$_{pr}$*::gfp* and *myo-3*$_{pr}$*::* ^{mt}*gfp* worms. N=4 biologically independent experiments. Error bars mean +/- s.d (Two tailed Student's *t*-test). RFU – relative fluorescence units.

f. Transcript levels of green fluorescent protein (*gfp*) as determined by qRT-PCR in *myo-3*$_{pr}$*::* ^{mt}*gfp* and in *atfs-1(null)*;*myo-3*$_{pr}$*::* ^{mt}*gfp* worms. N=2 biologically independent experiments.

g. *hsp-6*$_{pr}$*::gfp* and *atfs-1*$^{(\Delta NLS)}$*;hsp-6*$_{pr}$*::gfp* worms raised on control or *spg-7*(RNAi). Scale bar 0.1 mm. N=3 biologically independent experiments with similar results.

h. Transcript levels of heat shock protein-6 (*hsp-6*) as determined by qRT-PCR in wildtype and *atfs-1*$^{(\Delta NLS)}$ worms raised on *spg-7*(RNAi). N=3 biologically independent

experiments. Error bars mean +/- s.d. (Two tailed Student's *t*-test). RFU – relative fluorescence units.

i. Immunoblots of wildtype and *atfs-1*$^{(\Delta NLS)}$ worms raised on control or *lonp-1*(RNAi). ATFS-1 (▸). N=3 biologically independent experiments with similar results.

j. TMRE staining of wildtype and *atfs-1*$^{(\Delta NLS)}$ worms expressing *myo-3*$_{pr}$*::*^{mt}*gfp*. Scale bar 10 μm. Skeleton-like binary backbone are presented to the right. N=3 biologically independent experiments with similar results.

Source data are provided as a Source Data file.
