# Supplementary material for: UPRmt scales mitochondrial network expansion with protein synthesis via mitochondrial import in Caenorhabditis elegans
Source: Nat Commun. 2021 Jan 20;12:479. doi: 10.1038/s41467-020-20784-y (PMC7817664; doi:10.1038/s41467-020-20784-y)
Supplement: Supplementary file 10 — Description of additional supplementary files [file 41467_2020_20784_MOESM10_ESM.docx]

Description of additional supplementary files

Title: Supplementary Data file 1.

Description: Transcripts encoding mitochondria-localized proteins and glycoysis components regulated by *atfs-1(et18).* N=3 biologically independent experiments. Table analyzed by Two tailed student *t* test.

Title: Supplementary Data file 2.

Description: Transcripts encoding mitochondrial-localized proteins and glycolysis components expressed less in atfs-1(null) relative to wildtype worms. N=3 biologically independent experiments. Table analyzed by Two tailed student t test.

Title: Supplementary Data file 3.

Description: Transcripts encoding mitochondria-localized proteins and glycoysis components regulated by atfs-1(et18) and atfs-1(null). N=3 biologically independent experiments. Table analyzed by Two tailed student t test.

Title: Supplementary Data file 4.

Description: Transcripts encoding OXPHOS and citric acid cycle regulated by atfs-1(null). N=3 biologically independent experiments. Table analyzed by Two tailed student t test.

Title: Supplementary Data file 5.

Description: Transcripts encoding OXPHOS and citric acid cycle regulated by atfs-1(et18). N=3 biologically independent experiments. Table analyzed by Two tailed student t test.

Title: Supplementary Data file 6.

Description: Primers used in this study

Title: Supplementary Data file 7.

Description: Differentially expressed genes in atfs-1(et18) relative to wildtype worms. N=3 biologically independent experiments. Table analyzed by Two tailed student t test.

Title: Supplementary Data file 8.

Description: Differentially expressed genes in atfs-1(null) relative to wildtype worms. N=3 biologically independent experiments. Table analyzed by Two tailed student t test.
