# Supplementary material for: UPRmt scales mitochondrial network expansion with protein synthesis via mitochondrial import in Caenorhabditis elegans
Source: Nat Commun. 2021 Jan 20;12:479. doi: 10.1038/s41467-020-20784-y (PMC7817664; doi:10.1038/s41467-020-20784-y)
Supplement: Supplementary file 11 — Reporting Summary [file 41467_2020_20784_MOESM11_ESM.pdf]

nature research

Corresponding author(s): Cole Haynes

Last updated by author(s): December 30, 2020

Reporting Summary

Nature Research wishes to improve the reproducibility of the work that we publish. This form provides structure for consistency and transparency in reporting. For further information on Nature Research policies, see Authors & Referees and the Editorial Policy Checklist.

Statistics

For all statistical analyses, confirm that the following items are present in the figure legend, table legend, main text, or Methods section.

n/a	Confirmed	
☐	☒	The exact sample size (*n*) for each experimental group/condition, given as a discrete number and unit of measurement
☐	☒	A statement on whether measurements were taken from distinct samples or whether the same sample was measured repeatedly
☐	☒	The statistical test(s) used AND whether they are one- or two-sided<br>*Only common tests should be described solely by name; describe more complex techniques in the Methods section.*
☒	☐	A description of all covariates tested
☒	☐	A description of any assumptions or corrections, such as tests of normality and adjustment for multiple comparisons
☐	☒	A full description of the statistical parameters including central tendency (e.g. means) or other basic estimates (e.g. regression coefficient) AND variation (e.g. standard deviation) or associated estimates of uncertainty (e.g. confidence intervals)
☐	☒	For null hypothesis testing, the test statistic (e.g. *F*, *t*, *r*) with confidence intervals, effect sizes, degrees of freedom and *P* value noted<br>*Give P values as exact values whenever suitable.*
☒	☐	For Bayesian analysis, information on the choice of priors and Markov chain Monte Carlo settings
☒	☐	For hierarchical and complex designs, identification of the appropriate level for tests and full reporting of outcomes
☒	☐	Estimates of effect sizes (e.g. Cohen's *d*, Pearson's *r*), indicating how they were calculated

Our web collection on statistics for biologists contains articles on many of the points above.

Software and code

Policy information about availability of computer code

Data collection	Images were collected with the Zen2.3 blue edition microscoope from Zeiss. Blots were taken with Bio-Rad molecular imager ChemiDoc XRS+. Radiolabeled gels were scanned using a Typhoon Trio scanner (Amersham).
Data analysis	Blots and images were analyzed via Fiji (version 2.0.0-rc-69/1.52p). GraphPad PRISM 8 software was used to create the graphs. GSEA was performed with GSEA 3.0 software. Ribosome profiling data were analyzed with FastQC (version 0.11.9), BWA (Version 0.7.5), SAMtools (version 0.1.19) and data was plotted using R (Version 3.5.2).

For manuscripts utilizing custom algorithms or software that are central to the research but not yet described in published literature, software must be made available to editors/reviewers. We strongly encourage code deposition in a community repository (e.g. GitHub). See the Nature Research guidelines for submitting code & software for further information.

Data

Policy information about availability of data

All manuscripts must include a data availability statement. This statement should provide the following information, where applicable:

- Accession codes, unique identifiers, or web links for publicly available datasets
- A list of figures that have associated raw data
- A description of any restrictions on data availability

The raw data for the deep seq analysis that appears in supplementary tables 1-5 and supplementary tables 7-8 is publicly available. The data reported in this paper have been deposited in the Gene Expression Omnibus (GEO) database, [https://www.ncbi.nlm.nih.gov/geo/query/acc.cgi?acc=GSE114951] (accession no. GSE114951). Data also available from the corresponding author upon reasonable request. Source data are provided with this paper.

Field-specific reporting

Please select the one below that is the best fit for your research. If you are not sure, read the appropriate sections before making your selection.

☒ Life sciences ☐ Behavioural & social sciences ☐ Ecological, evolutionary & environmental sciences

For a reference copy of the document with all sections, see nature.com/documents/nr-reporting-summary-flat.pdf

Life sciences study design

All studies must disclose on these points even when the disclosure is negative.

Sample size

Sample sizes were not predetermined based on statistical methods. Sample sizes were based on previous experiments that yielded consistent and reproducible results in different worm strains.
1. All hsp-6pr::GFP imaging experiments were blindly tested via fluorescent microscopy for more than 100 worms per strain per condition per experiment before images were taken. Sample size was not determined because all the worms under each condition showed similar phenotypes. A Representative result from 3 biological independent experiments are presented. All experiments had consistent results.
2. For qPCR mRNA quantification and for deep seq experiments, approximately ~2000 worms were used per strain per condition per experiment.
3. For mtDNA quantification (30 worms were used per strain per experiment). From our pre-testing we obtain reproducible and consistent mtDNA quantification results from N>10 worms.
4. For developmental assays, at least 150 worms were tested per experiment. Experiments were repeated 2 biologically independent times with similar results.
5. Seahorse experiments included 90 worms (10 worms per well) and repeated 3 biologically independent times with similar results. Sample size was chosen according to: PMID: 27583642.
6. For ChIP experiments, approximately 3e+04 gravid adults were bleached and the progeny approximately 3e+06) was used per strain per experiment.
7. For TMRE intensity, at least 10 worms were analyzed per strain per condition per experiment.
8. For western blot analysis, approximately ~2000 worms were used per strain per condition per experiment.
9. For EM analysis we analyzed 5 different worms from each strain.

Data exclusions

No data was excluded from the analysis

Replication

All experiments were performed at least three times yielding similar results and comprised of biological replicates except for developmental assays, the null GFP qPCR and the ChIP. The results from the null GFP qPCR and the ChIP yielded similar results.

Randomization

For all experiments worms were pooled and randomly allocated to different plates and treatments.

Blinding

Blind group allocation was done for: Intensity measurements, fluorescent images and developmental assays as they may be subjected to human error. All other experiments were not blinded as they rely on objective instrument measurements such as for the qPCR analysis, deep seq and seahorse experiments.

Reporting for specific materials, systems and methods

We require information from authors about some types of materials, experimental systems and methods used in many studies. Here, indicate whether each material, system or method listed is relevant to your study. If you are not sure if a list item applies to your research, read the appropriate section before selecting a response.

Materials & experimental systems

n/a	Involved in the study
☐	☒ Antibodies
☐	☒ Eukaryotic cell lines
☒	☐ Palaeontology
☐	☒ Animals and other organisms
☒	☐ Human research participants
☒	☐ Clinical data

Methods

n/a	Involved in the study
☒	☐ ChIP-seq
☒	☐ Flow cytometry
☒	☐ MRI-based neuroimaging

Antibodies

Antibodies used

Antibodies against α-tubulin were purchased from Calbiochem (CP06). GFP and NDUFS3 from Abcam (ab6556 and ab14711 respectively). Antibodies for ATFS-1 were previously described PMID: 22700657. All antibodies were diluted 1:2000 except for ATFS-1 which was diluted 1:1000.

Validation

Validation for the ATFS-1 antibody was described previously: PMID: 22700657.

Validation statement from the company:Tubulin (CP06) Recognizes the ~60 kDa tubulin protein found in all eukaryotic cells.
NDUFS3(ab14711): validated in 23671424 and 27610574
GFP (ab6556)- validated in 27506200

Eukaryotic cell lines

Policy information about cell lines

Cell line source(s)	HEK293 cells - Human embryonic kidney. From ATCC.
Authentication	Cell lines were authenticated by morphology
Mycoplasma contamination	All cell lines were confirmed negative for mycoplasma contamination.
Commonly misidentified lines (See ICLAC register)	*Name any commonly misidentified cell lines used in the study and provide a rationale for their use.*

Animals and other organisms

Policy information about studies involving animals; ARRIVE guidelines recommended for reporting animal research

Laboratory animals	L4 hermaphrodites of the roundworm Caenorhabditis elegans were used in this study, except for supplementary figure 1b and supplementary figure 2b-d were L3 hermaphrodites were used.
Wild animals	This study did not involve wild animals
Field-collected samples	This study did not involve samples collected from the field.
Ethics oversight	No ethical approval was required.

Note that full information on the approval of the study protocol must also be provided in the manuscript.
